# Molecular recognition and maturation of SOD1 by its evolutionarily destabilised cognate chaperone hCCS

**DOI:** 10.1371/journal.pbio.3000141

**Published:** 2019-02-08

**Authors:** Fernanda A. Sala, Gareth S. A. Wright, Svetlana V. Antonyuk, Richard C. Garratt, S. Samar Hasnain

**Affiliations:** 1 Molecular Biophysics Group, Institute of Integrative Biology, Faculty of Health and Life Sciences, University of Liverpool, Liverpool, United Kingdom; 2 Instituto de Química de São Carlos, Universidade de São Paulo, São Carlos, Brazil; 3 Instituto de Física de São Carlos, Universidade de São Paulo, São Carlos, Brazil; Georgia Institute of Technology, UNITED STATES

## Abstract

Superoxide dismutase-1 (SOD1) maturation comprises a string of posttranslational modifications which transform the nascent peptide into a stable and active enzyme. The successive folding, metal ion binding, and disulphide acquisition steps in this pathway can be catalysed through a direct interaction with the copper chaperone for SOD1 (CCS). This process confers enzymatic activity and reduces access to noncanonical, aggregation-prone states. Here, we present the functional mechanisms of human copper chaperone for SOD1 (hCCS)–catalysed SOD1 activation based on crystal structures of reaction precursors, intermediates, and products. Molecular recognition of immature SOD1 by hCCS is driven by several interface interactions, which provide an extended surface upon which SOD1 folds. Induced-fit complexation is reliant on the structural plasticity of the immature SOD1 disulphide sub-loop, a characteristic which contributes to misfolding and aggregation in neurodegenerative disease. Complexation specifically stabilises the SOD1 disulphide sub-loop, priming it and the active site for copper transfer, while delaying disulphide formation and complex dissociation. Critically, a single destabilising amino acid substitution within the hCCS interface reduces hCCS homodimer affinity, creating a pool of hCCS available to interact with immature SOD1. hCCS substrate specificity, segregation between solvent and biological membranes, and interaction transience are direct results of this substitution. In this way, hCCS-catalysed SOD1 maturation is finessed to minimise copper wastage and reduce production of potentially toxic SOD1 species.

## Author summary

Cellular complexity necessitates an equally complex network of courier proteins to internalise, sort, and deliver biologically useful metals like copper. These relay systems negotiate a landscape of metal-binding sites through handshake–handoff interactions, but the mechanisms that impart a necessary transience are often not clear. Superoxide dismutase-1 (SOD1) is one of the most abundant human proteins and is an important part of our antioxidant, redox signalling and respiratory control mechanisms. If newly synthesised SOD1 is not correctly processed by the addition of copper, zinc, and an unusual disulphide bond, it will remain inactive or can misfold, as is the case in some neurodegenerative diseases. Here, we discover the mechanisms that govern SOD1 maturation and stabilisation through interaction with the chaperone protein hCCS. Conservation of our proposed mechanism across eukaryotes indicates it developed very soon after the gene duplication event that separated SOD1 and CCS coding sequences. SOD1 stability appears to have been quickly traded at the expense of CCS following the dawn of eukaryotic life, in order to efficiently produce this important enzyme.

## Introduction

Copper binding and disulphide bond formation are strongly discouraged in the eukaryotic cytoplasm despite widespread use within extracellular spaces and organelles. In the latter case, the preponderance of reduced glutathione and the thioredoxin system almost completely prevent their existence. In the former case, copper concentration is minimised to prevent adventitious binding or toxic chemistry [1]. However, production of superoxide dismutase-1 (SOD1) requires sequential copper and intra-subunit disulphide bond acquisition by a zinc-loaded precursor [2,3]. SOD1 folding, zinc and copper acquisition, and disulphide bond formation can be catalysed by the copper chaperone for SOD1 (CCS), which forms a transient heterodimeric complex with SOD1 to facilitate its maturation [4–8].

CCS is thought to retrieve copper primarily from membrane-bound sources, including direct transfer from copper transporter-1 (Ctr1) or indirectly through Atox1 and glutathione [9–12]. It then interacts with a pool of pre-existing SOD1 and selects the zinc-metalated, disulphide-reduced SOD1 substrate from at least 16 possible other states [5,13]. The molecular recognition event that dictates CCS specificity is a fulcrum point for the efficient management of intracellular copper, maintenance of an adequate antioxidant response, and redox signalling but also helps to avoid accumulation of aggregation-prone immature human SOD1 (hSOD1) [14,15] (S1A Fig). Indeed, the human copper chaperone for SOD1 (hCCS) activates at least 80% of hSOD1 molecules [16]. The harmful effects of incomplete hSOD1 maturation are clearly seen in the motor system diseases amyotrophic lateral sclerosis (ALS) and possibly Parkinson disease [17,18]. Typically of neurodegenerative disease, reduced stability of hSOD1 potentiates formation of toxic oligomers [19], aggregation [20], and irretrievable sequestration into distinct cytoplasmic compartments [21,22]. Once posttranslation modification (PTM) transfer processes are complete, hCCS must disengage from hSOD1. Transience of the interaction is paramount, as hSOD1 must homodimerise for full activity and stability [23], but it is not clear how that ephemerality has been engineered into the system.

**Fig 1:**
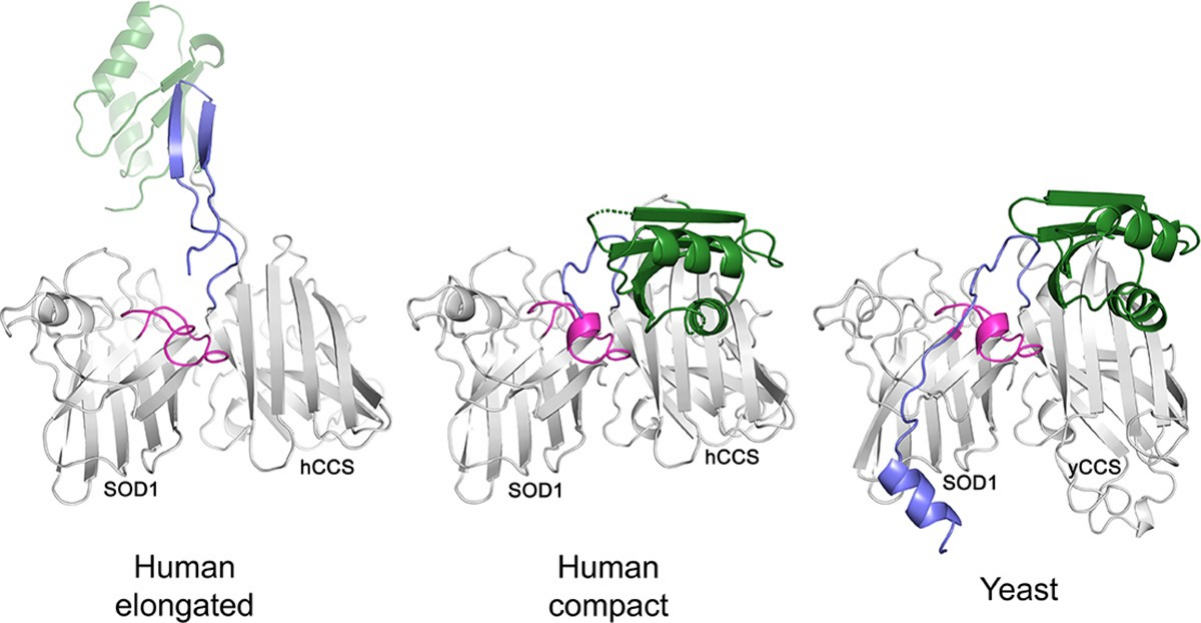
Comparison of human and yeast CCS-SOD1 complex structures. The human CCS-SOD1 complex is presented in two conformations, elongated (6FON) and compact (6FP6) and is compared with the structure of yeast CCS-SOD1 (1JK9). Relevant domains and loops are coloured: SOD1 disulphide sub-loop, pink; CCS domain I, green; CCS C-terminal domain, mauve. For a complete breakdown of structures and mutations presented, please see S1B Fig. CCS, copper chaperone for SOD1; SOD1, superoxide dismutase-1.

Here, we describe several structures of the hSOD1 activating complex crystallised by inhibiting complex dissociation and aggregation through discerning mutagenesis of cysteines involved in normal and aberrant disulphide bond formation. Combinations of mutants yielded crystallographic structures of full-length hCCS in two different conformers complexed with hSOD1; an hCCS domain II truncation complexed with hSOD1; an hCCS domain II homodimer structure at higher resolution than previously available (1.55 Å); and the hSOD1 disulphide knock-out mutant used to promote complexation (Fig 1, S1B Fig and S1 Table). These snapshots of reaction precursors, intermediates, and products are important landmarks on a journey through a transient interaction that has critical importance in the maintenance of normal metabolic processes, including regulation of respiration. We find the functionality of the complex is driven by an evolutionarily fine-tuned affinity gradient. The initial molecular recognition and complexation event imposes a structure on hSOD1 and facilitates sequential copper and disulphide PTMs. At the core of this system, a single methyl group resulting from the conserved substitution of alanine for glycine within the hCCS dimer interface orchestrates sequential steps in the folding and PTM pathway to produce stable and active hSOD1.

## Results and discussion

### hCCS is destabilised by an interface methyl group

**Fig 2:**
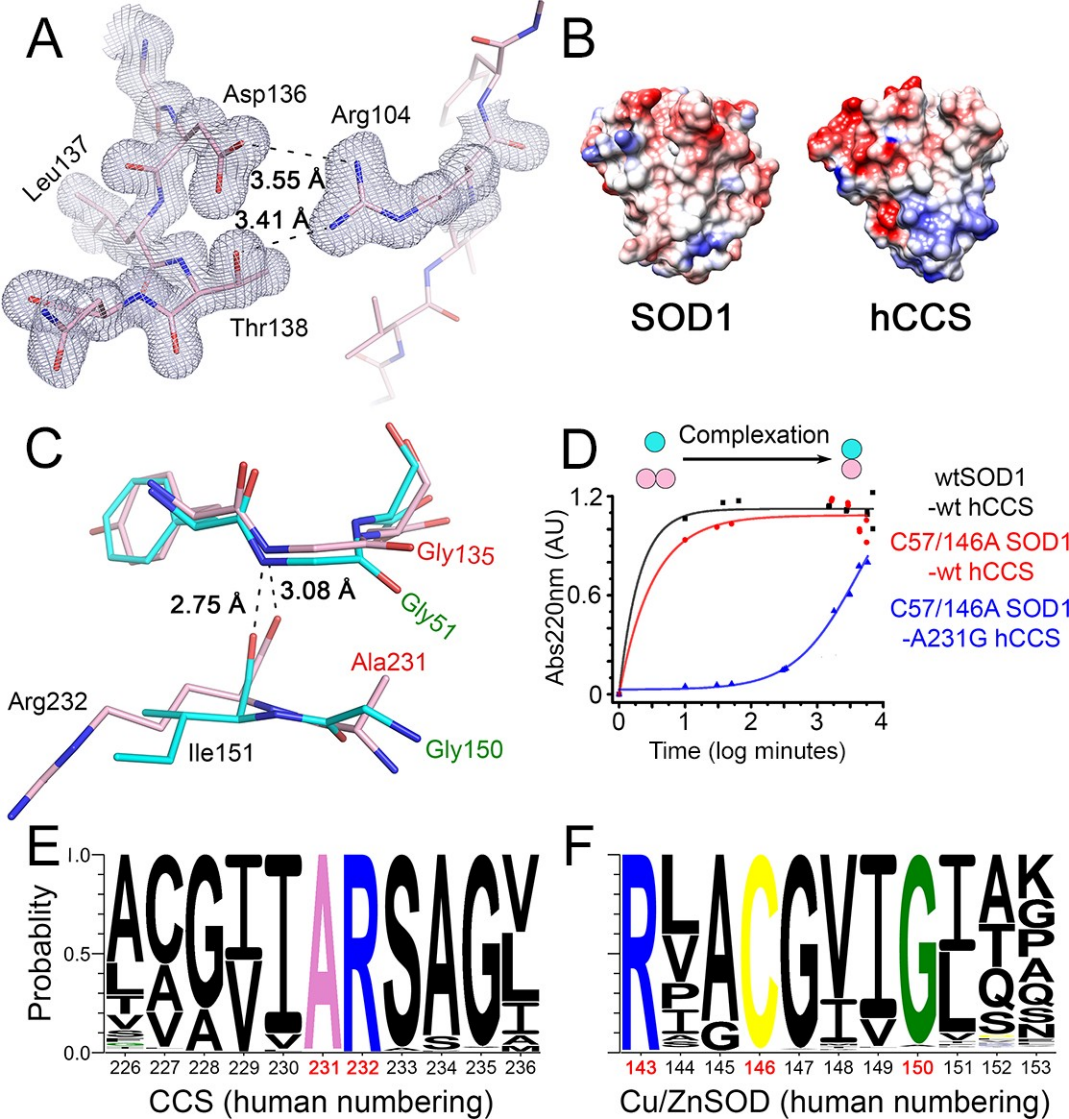
hCCS dimer interface destabilisation. (**A**) Electron density map (2Fo-Fc, contoured at 1σ level) showing the Coulombic interaction between hCCS Arg104 and Asp136/Thr138. (**B**) Surface charge maps of hSOD1 and hCCS domain II dimer interface surfaces. (**C**) The hCCS (pink) dimer interface Arg232-Gly135 hydrogen bond is weakened by the steric effect of Ala231 side chain. The hCCS Gly135-Asp136 carbonyl rotates to accommodate the methyl group, and Gly135 is pushed away from Arg232. hSOD1 (cyan) Gly150 maximises hydrogen bond strength between Phe51 and Ile151. (**D**) Restoring SOD1-like dimer affinity with the Ala231Gly hCCS mutation vastly slows complexation. (**E**) Eukaryotic CCS sequence diversity shows Ala231 is very highly conserved despite its detrimental effect on homodimer affinity. (**F**) SOD1 Gly150 is equally well conserved, indicating the relative balance of SOD1 and CCS homodimer affinities is evolutionarily static. AU, absorbance unit; CCS, copper chaperone for SOD1; hCCS, human copper chaperone for SOD1; hSOD1, human superoxide dismutase-1; SOD1, superoxide dismutase-1; wt, wild-type.

Molecular recognition and affinity are dictated by binding free energy. When homodimeric hSOD1 and hCCS domain II are physiologically zinc metalated, many of the terms that comprise binding free energy are equal due to their sequence and structural similarity. Indeed, orientation of monomers and the presence of four inter-subunit hydrogen bonds found in homodimeric hSOD1 are maintained by hCCS (S1B Fig, S2A and S2B Fig, S2 and S3 Table). While fully mature hSOD1 has low nanomolar affinity homodimer affinity [24], zinc-metalated, disulphide-reduced hSOD1 has a dimer dissociation constant of 51 μM [25]. Thus, within cells, the majority of hCCS’s substrate will be in a monomeric state. It is axiomatic that hCCS has a higher affinity for this species than for itself, but given mature hCCS forms SOD1-like homodimers, the question remains, why? The presence of a stabilising Coulombic interaction between opposing hCCS Arg104 and Asp136 residues (3.55 Å) (Fig 2A), which is not present in hSOD1, appears counterintuitive in this regard. However, the hCCS dimer interface is replete with positive charge and therefore not dominated by the hydrophobic effect seen in hSOD1 and many other protein complexes (Fig 2B).

Most importantly, however, the presence of hCCS Ala231 significantly weakens half of the homodimer interface hydrogen bonds conserved from SOD1. Specifically, Ala231 side-chain methyl pushes Arg232 and Gly135 apart through steric repulsion, lengthening the carbonyl-amine hydrogen bond between them to 3.08 Å from 2.75 Å, in the case of the homologous SOD1 residues Ile151 and Gly51 (Fig 2C). Without this rearrangement, hCCS Ala231 Cβ would be an energetically unfavourable 2.3 Å from the Gly135 carbonyl oxygen. Introduction of the hCCS Ala231Gly amino acid substitution to mimic the hSOD1 dimer interface increases hCCS dimer affinity (S2D Fig), slows complexation with hSOD1 more than 300-fold (Fig 2D), impedes hSOD1 activation (S2E Fig), and hSOD1 disulphide formation (S2F Fig). Glycine and alanine are near ubiquitous at these positions in eukaryotic copper/zinc superoxide dismutases (Cu/ZnSODs) and their cognate chaperones, respectively (Fig 2E and 2F). Thus, relatively weak CCS homodimer affinity has been evolutionarily maintained to provide a pool of monomeric CCS available to interact with and activate nascent monomeric SOD1 within a physiologically relevant timescale. An interesting exception is the nematode CuZnSODs, which have a deforming alanine in place of hSOD1 Gly150 but are exclusively activated by a CCS-independent means [26].

### Evolutionarily conserved induced-fit complexation

**Fig 3:**
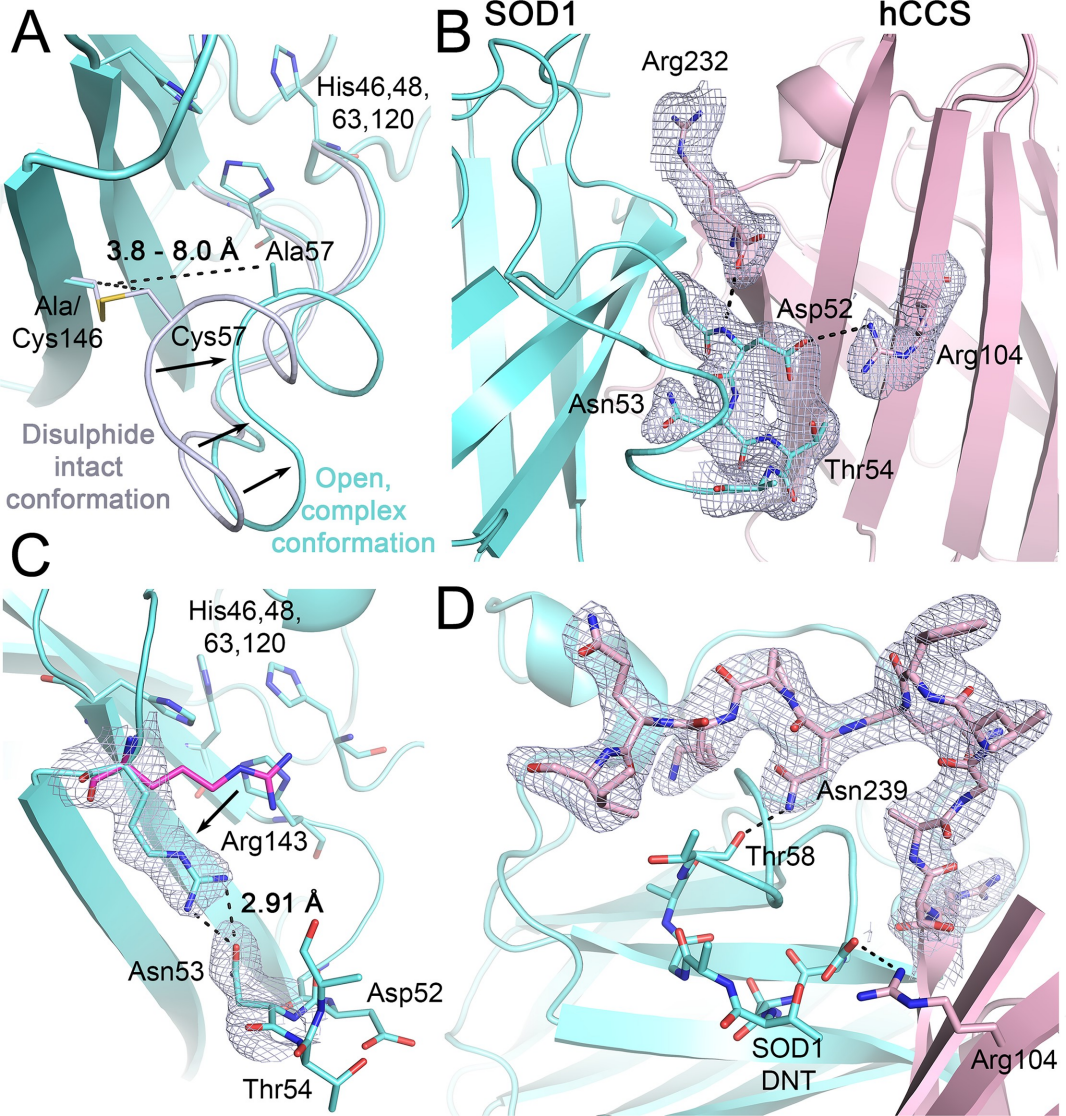
hSOD1 induced-fit complexation. (**A**) Complexation with hCCS forces the hSOD1 disulphide sub-loop to adopt an open conformation separating the amino acids involved in disulphide formation. (**B**) Interactions across the heterodimer interface stabilise the hSOD1 disulphide sub-loop. (**C**) The hSOD1 Arg143 side chain interleaved between the β-barrel and disulphide sub-loop, hydrogen bonded with the DNT motif Asn53, and compared with the conformation found in homodimeric hSOD1 (magenta). (**D**) hCCS domain III is recruited to the hSOD1 disulphide sub-loop by a restrictive domain I position and hydrogen bonding between hCCS C-terminal Asn239 and hSOD1 Thr58. This brings the functional hCCS CXC motif into the vicinity of hSOD1 Cys57. 2Fo-Fc electron density maps are contoured at the 1σ level. hSOD1 is shown in cyan, hCCS in pink. CXC, Cys-Xxx-Cys; GDNT, Gly51-Asp52-Asn53-Thr54; hCCS, human copper chaperone for SOD1; hSOD1, human copper chaperone for SOD1; SOD1, superoxide dismutase-1.

The SOD1-like, hCCS domain II found within each complex structure has an intact intra-subunit disulphide bond in contrast to the yeast orthologue [27] (S3A Fig). This disulphide does not dictate complexation (S3B Fig) but does thermally stabilise the hCCS homodimer and the complex with hSOD1 (S3C and S3D Fig). In contrast, hSOD1 must be in the disulphide-reduced state to complex with hCCS. We recently predicted hSOD1 disulphide sub-loop (amino acids His48–His63) movement on complexation with hCCS based on small-angle x-ray scattering (SAXS) data [28,29]. This is proven true by our new structures, where it adopts a conformation that could not be accommodated if the hSOD1 disulphide were present (Fig 3A). This conformation is not found in mutant or wild-type hSOD1 disulphide intact dimer [30], Cys57/146Ala disulphide knock-out dimer, or mutation-dependent obligate monomer structures [31,32] despite the extensive conformational sampling present (S4A–S4I Fig). In illustration of this point, a recent nuclear magnetic resonance (NMR) characterisation indicated SOD1 amino acids 49–54 are disordered in the zinc-metalated, disulphide-reduced state [25]. Conversely, human and yeast SOD1 adopt an identical sub-loop conformation when complexed with yeast CCS [12,33]. Without the Cys57-Cys146 covalent tether, the hSOD1 disulphide sub-loop can adapt to the presence of hCCS Ala231, mitigating repulsive effects. As a result, several strong hydrogen bonds are formed across the heterodimer interface that are not found in the SOD1 homodimer (S4 Table). Particularly important is the hydrogen bond/salt bridge network between hCCS Arg104/Arg232 and opposing hSOD1 Asp52 (Fig 3B). In this way, hCCS provides a surface of repulsive and attractive noncovalent interactions that mould the plastic hSOD1 disulphide sub-loop into a stable but novel conformation. This provides a mechanism for the molecular chaperone activity of hCCS [7]. The SOD1 disulphide sub-loop is also stabilised by several internal hydrogen bonds not found in the mature enzyme (S4J Fig and S5 Table). An accumulation of these effects separates Cβ carbons of residues 57 and 146 by 8.0 Å (Fig 3A). Rotation of the alanine residue, here replacing Cys57, orientates this functional side chain like a flagpole marking the entrance to the hSOD1 active site. Thus, hCCS-SOD1 molecular recognition and complexation proceed by an induced fit mechanism reliant on the conformational adaptability of the hSOD1 disulphide sub-loop and hCCS domain II only. We suggest this primes hSOD1 for reception of copper and the disulphide, before any interaction with hCCS domain I or III takes place.

The SOD1 disulphide sub-loop Gly51-Asp52-Asn53-Thr54 (GDNT) tetrad motif forms many of the interactions across the hSOD1-hCCS heterodimer interface. It is evolutionarily conserved in eukaryotic Cu/ZnSOD enzymes, with the β-barrel–facing Asn53 substituted by a variety of amino acids, including threonine, leucine and serine; Gly51-Asp52-Xxx-Thr54 (GDXT) (S5A Fig). Disruption of the hCCS Arg104-GDNT interaction by the human hSOD1 substitution Thr54Arg inhibits hCCS-catalysed disulphide formation and is causative for a subset of ALS [12]. A GDXT/S tetrad is also present in hCCS and its orthologues (S5B Fig). Negation of the Arg104-GDNT interaction with an hCCS R104A mutation does not inhibit complex formation or hSOD1 thiol oxidation and activation but destabilises the hCCS homodimer and the hCCS-SOD1 complex, introducing a low-temperature melting transition (S2E, S2F and S5C Figs). hCCS Arg104 is near ubiquitously conserved among CCS orthologues (S5B Fig) with the only exception being the cetacean conservative mutation to histidine (S5D Fig), which also induces a low-temperature melting transition for both hCCS homodimer and hCCS-SOD1 heterodimer (S5C and S5E Fig). Together, this indicates that the length and charge of the Arg104 side chain is important for structural stability in homodimeric or heterodimeric states as a consequence of the distance between hCCS β-strand 2 and the opposing disulphide sub-loop motif.

### Intermolecular communication determines the timing of PTM transfer

If the hSOD1 disulphide is formed before copper is passed from hCCS, the complex will dissociate, leaving the hSOD1 product inactive. Thus, PTM transfer events must be correctly sequenced. The hSOD1 active site is formed in part by the Arg143 side chain, which directs superoxide to the copper centre and hydrogen bonds with the substrate during catalysis [34]. The conformation of the Arg143 side chain is sensitive to the position of residue 57 and therefore the propensity of hSOD1 to form a homodimer [31,34] (S4A and S4B Fig). When hSOD1 complexes with hCCS its copper site is exposed to solvent by a shift in the Arg143 side chain (Fig 3C). This is due to inability of the Arg143 guanidinium group to hydrogen bond with Gly61 and Cys57 carbonyls found within the disulphide sub-loop, due to increased distance (3.0 to 3.5 Å). As a result, it interposes between residues 57 and 146 in the space normally occupied by the hSOD1 disulphide. In this conformation, the Arg143 guanidinium hydrogen bonds with the hSOD1 disulphide sub-loop GDNT tetrad Asn53. We propose that this effect is an integral part of the activation mechanism; communication from hCCS via Arg104 through the hSOD1 GDNT motif to the Arg143 side chain ensures the amenability of the active site to receive copper in response to complexation with hCCS. Simultaneously, Arg143 forms a physical barrier between disulphide bonding residues and occludes the electropositive cavity found when hSOD1 is complexed with mutant yeast CCS [12]. Arg143 side-chain movement has been observed in the yeast CCS-SOD1 complex, where it is found hydrogen bonded to the alanine amide of the yeast CCS (yCCS) Cys-Xxx-Cys (CXC) C-terminal motif [33]. Copper transfer or recruitment of the hCCS CXC motif therefore switches the Arg143 side chain removing a potential block to disulphide formation. This mechanism would prioritise copper transfer over disulphide formation so that interactions that yield inactive or unstable SOD1 product are minimised.

From a conformation that facilitates copper acquisition from Ctr1/Ctr2 or Atox1, hCCS domain I must move to a position that enables transfer to hSOD1. The very high positional dynamism necessary of the hCCS copper-binding domain is evidenced by intra-lattice conformational flexibility and a 47.4-Å domain movement between conformers (S6A and S6B Fig). Conformational plasticity is therefore not restricted to the hCCS homodimer state [35] but is an intrinsic property of the activating complex. The act of copper transfer between hCCS domain I and hSOD1 is driven by the higher affinity of the hSOD1 tetrahistidine site compared with the domain I bis-cysteine site and facilitated by intermediate chelating side-chain interactions from the hCCS CXC motif and hSOD1 Cys57 within the disulphide sub-loop [12,36]. Comparison of different hCCS conformers indicate that the C-terminal tail is also conformationally dynamic, as has been observed for the yeast orthologue [12,33] and predicted from SAXS data for hCCS [35]. When hCCS domain I is free to move within the lattice or inhabits the extended conformer, the position of domain III is partially or entirely unrestricted. In the latter case, it forms the interface of a supramolecule comprising four hCCS monomers and four hSOD1 monomers in crystallo (S6C Fig). By contrast, when domain I is positioned close to the substrate hSOD1 molecule in the compact conformer, it stabilises domain III by restricting space and forming a series of conserved interdomain hydrogen bonds (S6D and S6E Fig). As a result, domain III arches over the hSOD1 disulphide loop and forms a side-chain hydrogen bond between hCCS Asn239 and hSOD1 Thr58 carbonyl, which can only exist when the hSOD1 disulphide sub-loop is in the induced fit conformation (Fig 3D and S6F Fig). Both the conformation and hydrogen bonding are again conserved and provide the impetus to bring the functionally important hCCS CXC motif into position next to Cys57. The noncovalently bonded C-terminal conformation existing in the compact structure presented here is therefore a precursor of the yeast mixed disulphide–bonded structure [33]. Thus, induced-fit SOD1 disulphide sub-loop conformation change upon complexation ultimately recruits the hCCS functional motifs necessary to ensure timely PTM transfer.

### A molecular lever affects complex dissociation

**Fig 4:**
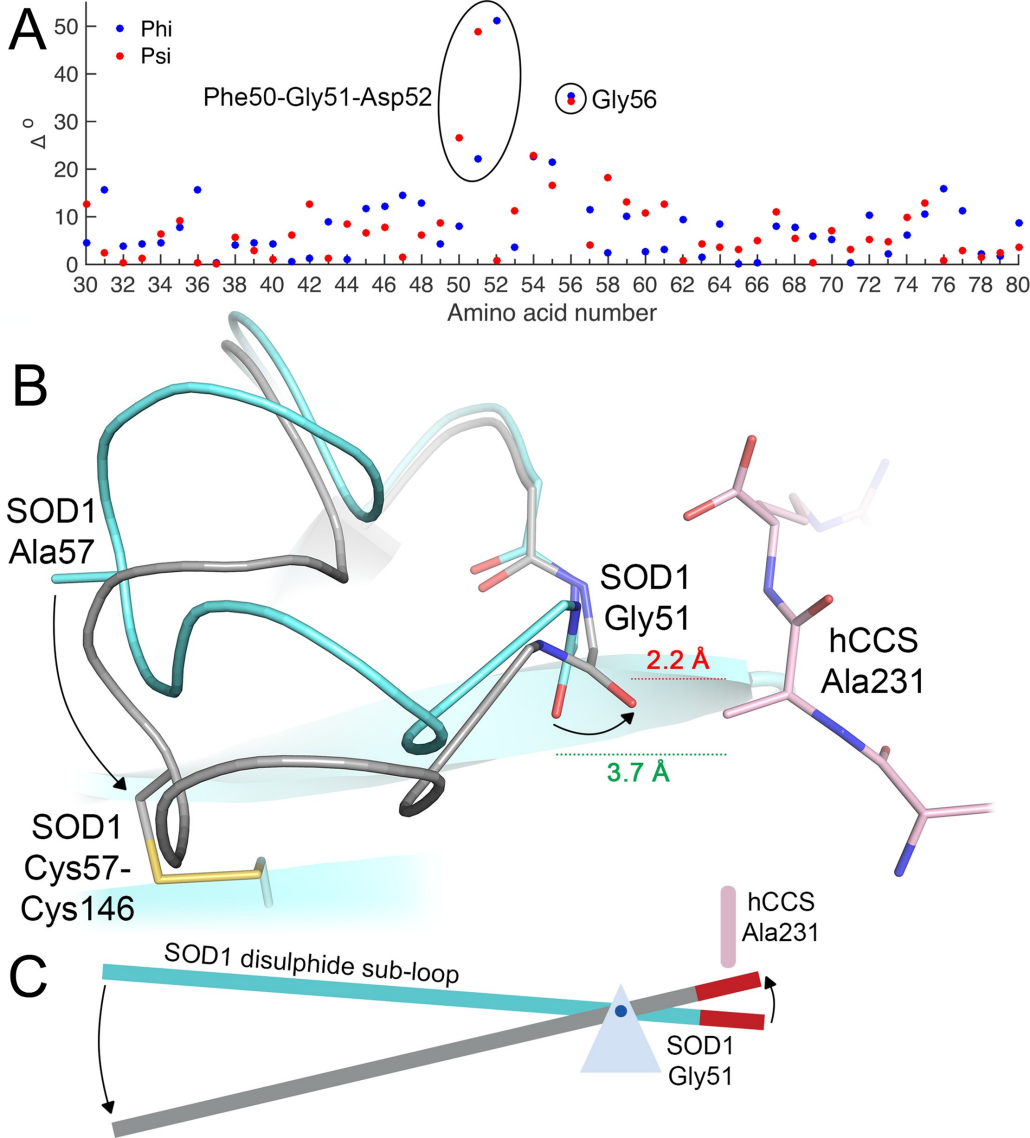
SOD1 Gly51 is the fulcrum of a molecular lever. (**A**) Dihedral and (**B**) conformational change on the formation of the SOD1 disulphide. Rotation around Gly51 pitches the loop toward the SOD1 β-barrel, forcing a steric clash with hCCS Ala231. (**C**) Represented schematically. hCCS, human copper chaperone for SOD1; SOD1, superoxide dismutase-1.

For the Cys57 and 146 sulphydryls to form the disulphide bond, the whole sub-loop pivots on Gly51, and a Cys57 orientation change is accommodated by Gly56 (Fig 4A). Here, the disulphide sub-loop operates as a class I lever forcing the Gly51 carbonyl too close to hCCS Ala231 and deforming the hSOD1 Gly51-hCCS Arg232 interface hydrogen bond (Fig 4B and 4C). hSOD1 then dimerises due to of mitigation of repulsive effects by substitution of Gly150 for hCCS Ala231, formation of the four strong interface hydrogen bonds, breaking of both Arg104- and Arg232-GDNT heterodimer noncovalent bonding interactions (S6 Table), loss of electrostatic repulsion as Arg232 is replaced by hSOD1 Ile151, and maximising the stable, hydrophobic interface surface. Thus, interactions between the GDNT disulphide sub-loop tetrad across the heterodimer interface dictate the specificity of hCCS for disulphide-reduced hSOD1, the timing of copper and disulphide transfer, and complex dissociation. The affinities that regulate these events are finely balanced as a necessity of the similarity of the proteins involved and the small disulphide loop conformation change that directs the interaction. Only 3% of the amino acids present dictate complex recognition, while the hCCS Ala231 methyl, which orchestrates complexation and dissociation, constitutes less than 0.04% of the total mass of the complex. In addition, while hSOD1 disulphide flexibility is viewed negatively as an aspect of the pathogenesis of hSOD1-related ALS and now possibly Parkinson disease [17,18], here we find that Gly51-pivoted sub-loop conformational switching is an absolute necessity for hCCS-catalysed hSOD1 activation.

### Copper retrieval by hCCS

**Fig 5:**
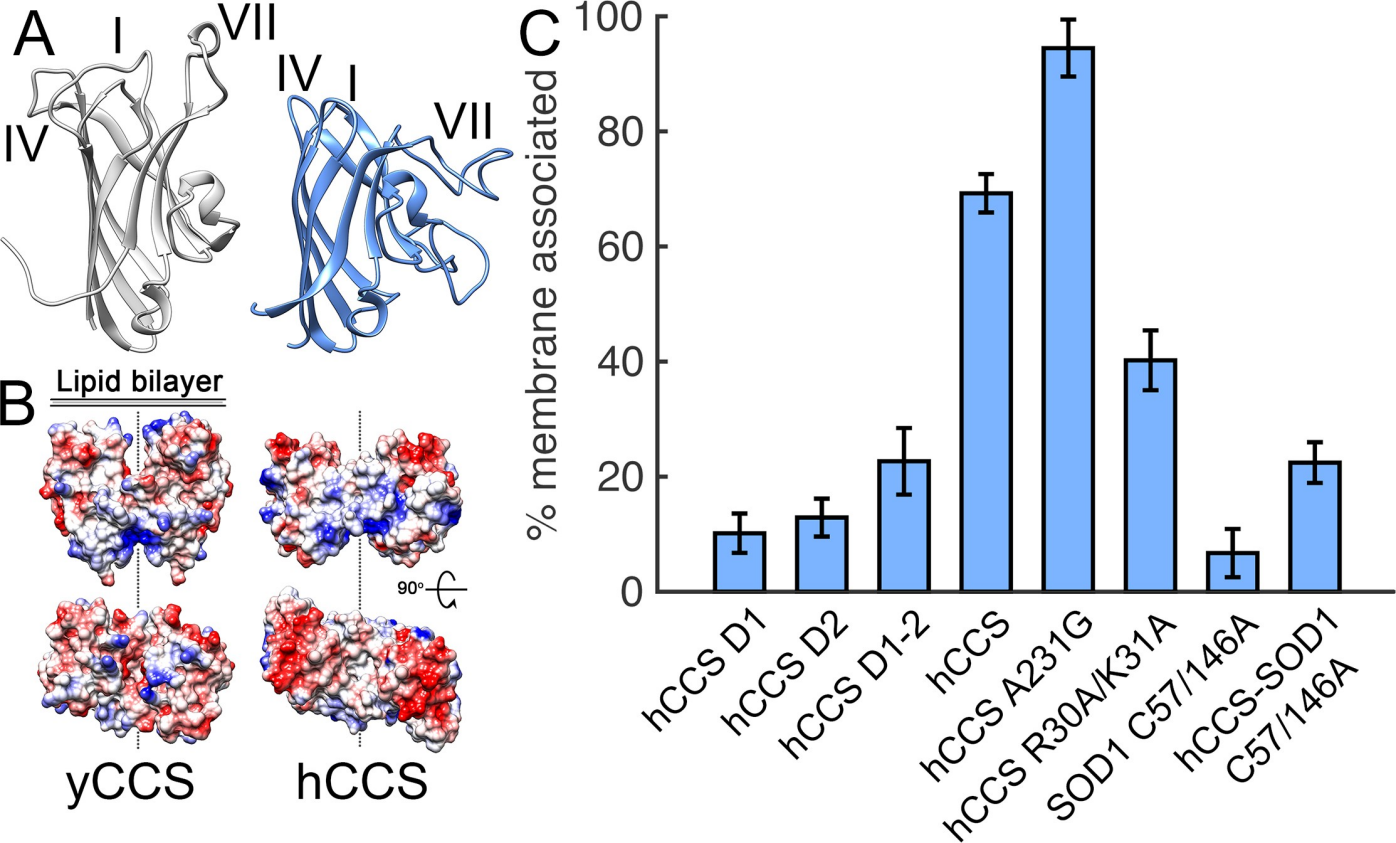
Dissimilar CCS membrane interaction surfaces. (**A**) The structure of domain II β-barrel loops I, IV, and VII are not conserved among yeast and human CCS orthologues. (**B**) Coulombic charge representation showing differing putative hCCS and yCCS domain II membrane-interacting surfaces. (**C**) Liposome-binding assay showing how the concerted effect of all three hCCS domains facilitate membrane association (S7C and S7D Fig). hCCS dimer affinity, domain I electropositivity, and complexation with SOD1 all effect lipid association. Mean ± SEM, *n* = 5. CCS, copper chaperone for SOD1; hCCS, human copper chaperone for SOD1; SOD1, superoxide dismutase-1; yCCS, yeast copper chaperone for SOD1.

While hCCS is 27% identical to yCCS, there are important differences in sequence, structure, and behaviour. Homodimeric hCCS and yCCS associate with negatively charged lipid bilayers representative of the inner surface of the plasma membrane. This is thought to minimise the spatial sampling necessary to locate membrane-bound copper sources [9]. Primary and secondary structure elements that facilitate membrane association are, however, not conserved from yeast to human CCS (Fig 5A and 5B and S7A Fig). Consequently, an hCCS domain II truncation does not strongly associate with lipids (Fig 5C). Despite similarities between hCCS domain I and Atox1 (S7B Fig), a monomeric hCCS domain I truncation also does not segregate significantly with liposomes (Fig 5C). When hCCS domain I and II are both present, they engender a stronger membrane association. This truncated protein exists in a monomer-dimer equilibrium at low micromolar concentration, with the majority as monomer (S7C Fig). An hCCS domain II–III construct has increased dimer affinity and exists as a dimer at micromolar concentrations (S7D Fig), but a positively charged patch in the conformationally plastic domain III (S7E and S7F Fig) does not aid association of hCCS to membranes. Increasing hCCS dimer affinity through the hCCS Ala231Gly mutation increases membrane interaction. Conversely, removing two positively charged Arg30 and Lys31 residues, which are sited close to the domain I copper site, decreases membrane association (Fig 5C). Thus, hCCS membrane association is mediated by the combination of globular domains I and II together with the increased interaction surface area provided by domain III–mediated dimerisation. While metal-free, disulphide-reduced wild-type hSOD1 associates with and even penetrates lipid membrane [37,38], on zinc binding, this association is greatly reduced (Fig 5C). Thus, the substrate for hCCS provides little additional membrane attraction, and half of the interfacial interacting surface provided by hCCS homodimerisation is lost on heterodimerisation. The hCCS-hSOD1 complex has little affinity for the lipid bilayer as a result (Fig 5C). Copper acquisition by hCCS is therefore likely to occur in the homodimeric state while membrane bound, and prior to complexation with hSOD1. Subsequent activation of hSOD1 is more likely to occur in solvent, off the bilayer.

**Fig 6:**
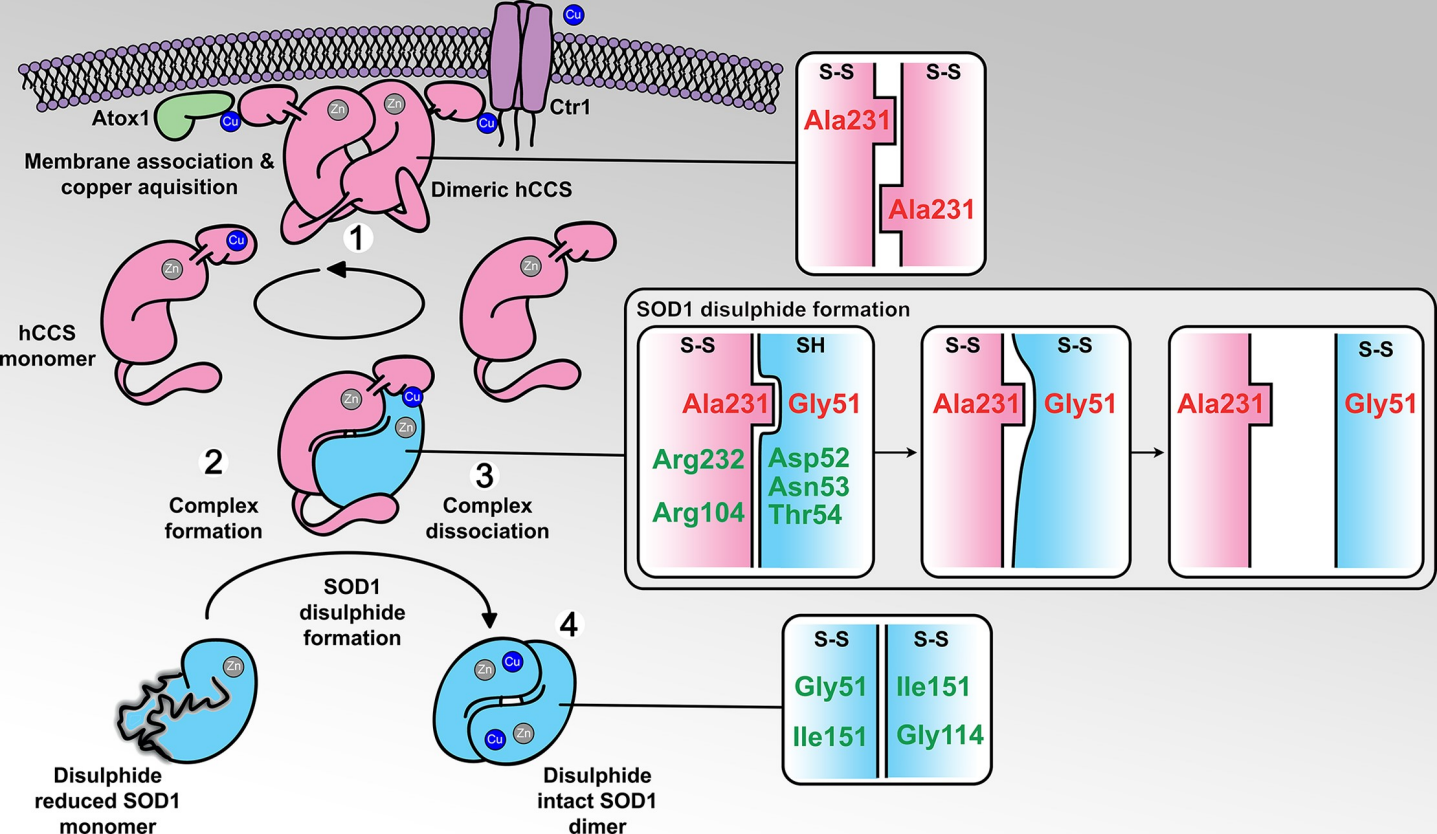
hCCS-catalysed hSOD1 activation. **1**. hCCS Ala231 facilitates a dynamic association with membranes while mobilising monomeric hCCS for complexation with hSOD1. **2**. Noncovalent interactions between hCCS domain II and the GDNT motif of the plastic hSOD1 disulphide sub-loop dictate molecular recognition. Complexation-induced conformational rearrangement prepares hSOD1 to receive copper, while impeding disulphide formation. **3**. hSOD1 disulphide formation breaks GDNT motif interactions, rotates the SOD1 disulphide loop, and weakens heterodimer affinity. **4**. hSOD1 forms a stable, active homodimer. hCCS can homodimerise and relocate back to the cytoplasmic membrane. hCCS, pink; hSOD1, blue. Panels show interactions across dimer interfaces, with disulphide status indicated as SH, reduced; S-S, intact. Ctr1, copper transporter-1; GDNT, Gly51-Asp52-Asn53-Thr54; hCCS, human copper chaperone for SOD1; hSOD1, human superoxide dismutase-1.

In summary, the synthesis of sequence analysis, biophysical assays, and long-awaited crystallographic structures of hCCS in multiple states have provided us with insight on the intricate mechanisms that catalyse assembly of stable and active SOD1. The weakened dimer affinity of hCCS resulting from Ala231 steric effects, its intracellular concentration, and the strength of lipid association appear finely tuned to establish a dynamic equilibrium that retrieves copper from membrane-bound sources and delivers it to membrane-free hSOD1 (Fig 6). A combination of repulsive and attractive interactions across the hCCS-SOD1 dimer interface assists SOD1 folding, prepares it for PTM acquisition, and dictates the timing of those modifications. Ultimately, complex dissociation is affected by a molecular lever operating with SOD1 Gly51 as its fulcrum, forcing an unfavourable steric clash and weakened hydrogen bonding across the dimer interface. Thus, SOD1 homodimerisation becomes energetically favourable, and it attains a stable, active state (Fig 6).

SOD1 and CCS appear to have diverged from their common ancestor very early in eukaryotic evolution, given the presence of both orthologues in almost all species. The near ubiquity of alanine in a destabilising position within the CCS dimer interface indicates that the finely balanced affinity gradient that drives human CCS-catalysed SOD1 maturation applies to all eukaryotic Cu/ZnSOD-chaperone pairs. It is not clear whether the CCS and SOD1 common ancestor harboured the stabilising glycine or destabilising alanine variation given the presence of the destabilizing variant in nematode CuZnSODs, however, the nonsynonymous mutation leading to this substitution happened very soon after the gene duplication event that separated SOD1 and CCS coding sequences. Evolution appears to have very quickly traded CCS stability for SOD1 stability. In great apes, including humans, this effect is particularly pronounced, with SOD1 having undergone strong positive selection to limit instability and thereby extend life span [39]. In contrast, a weakened CCS dimer interface does not appear to swamp cellular proteostasis machinery in the same way that SOD1 dimer interface destabilising mutations do [20,40], possibly due to reduced relative expression [41] or more efficient degradation. This adaptation, and the mechanism we propose, may be a critical milestone in the development of the large, highly compartmentalised forms into which eukaryotic cells have developed.

## Materials and methods

### Protein expression, purification, and complex formation

Protein expression, purification, and complex formation was performed as previously described [29,35,42], with the exception of SOD1 C57/146A in the pET3A vector, which was transformed into *Escherichia coli* BL21 (DE3), and expression was induced with 0.4 mM of IPTG with the addition of 0.2 μM ZnCl_2_ and incubated at 37°C for 6 hours.

### Crystallisation

All crystals were grown using the hanging-drop vapour diffusion method at 20°C from proteins in 20 mM *tris*(hydroxymethyl)aminomethane-HCl (Tris-HCl), pH 7.4, 150 mM NaCl, 1 mM dithiothreitol (DTT). hSOD1 C57/146A was crystallised from 1.0 μL of protein at 15 mg/mL, mixed in equal proportions with 0.2 M lithium sulphate; 0.1 M Tris-HCl, pH 8.0; and 24% w/v PEG 4000. Crystals appeared after 10 days of incubation. hSOD1 C57/146A− hCCS domain II at 15 mg/mL was crystallised in 25% (w/v) PEG 1500; 0.1 M PCTP buffer, pH 7.0 (sodium propionate, sodium cacodylate, and bis-tris propane in the molar ratios 2:1:2, respectively). Crystals grew after 15 days in space group H32, with four heterodimers in the asymmetric unit (ASU). Heterocomplexes, hSOD1(C57/146A)—full-length hCCS(C22/25S) (elongated conformer); hSOD1(C57/146A)—full-length hCCS(C12/22/25/244/246A) (compact conformer); and hCCS D2 were crystallised from nondiffracting seeds prepared from hSOD1(C57/146A)—full-length hCCS (C22/25S) crystals grown in 0.2 M sodium malonate, 20% (w/v) PEG 3350, frozen in the same solution in liquid nitrogen and stored at −80°C. For seed preparation, crystals were crushed and diluted serially: 1:5, 1:25, 1:125, and 1:625 in buffer dependent on the crystallisation condition with 20% (w/v) PEG 3350. Drop volume ratio consisted of 3 parts protein (1.2 μL):2 parts reservoir solution (0.8 μL):1 part stock (0.4 μL). For hSOD1(C57/146A)—hCCS(C22/25S) (elongated conformer), the complex was crystallised at 20 mg/mL in 0.1 M MES, pH 6.0, 0.2 M magnesium chloride, 20% (w/v) PEG 6000. Crystals appeared within 45 days. hSOD1(C57/146A)—full-length hCCS(C12/22/25/244/247A) (compact conformer) at 8 mg/mL was crystallised in 0.1 M PCTP buffer, pH 9.0, 20% (w/v) PEG 3350. Crystals appeared within 25 days. Crystals for hCCS domain II homodimer grew from 1.2 μL of 15 mg/mL full-length hCCS with the addition of 0.2 M sodium chloride, 20% (w/v) PEG 6000; 0.1 M HEPES, pH 7.0, reservoir solution after 8 months.

### Data collection and structure determination

All crystals were transferred into cryoprotective solution consisting of the respective reservoir solution and 20% glycerol and then flash frozen in liquid nitrogen. Data for all structures except C57/146A hSOD1 were collected at Soleil on beamline Proxima 1 with 0.97857 Å wavelength. C57/146A hSOD1 data were collected on Diamond beamline IO3 using 0.97626 Å wavelength. In all cases, a PILATUS 6 M detector was used. Images were integrated with iMosflm [43] or XDS [44] and scaled with SCALA [45] or AIMLESS [46]. All structures were solved by molecular replacement using PHASER [47] or MOLREP [48]. hCCS domain II homodimer and SOD1 C57/146A–hCCS domain II used hCCS and SOD1 structures 1DO5 and 2CV9, respectively, as the search model. Full-length forms were solved using a hSOD1 C57/146A–hCCS domain II heterodimer structure. The structures presented were constructed with successive rounds of manual model building in COOT [49] and refinement with a combination of Phenix [50] and Refmac [51]. Structures were validated with PDB validation tool and deposited in the Protein Data Bank with accession codes 6FOI, 6FN8, 6FOL, 6FON, and 6FP6.

### Thermal stability

Thermal stability was assayed by differential scanning fluorometry with a protein concentration of 10 μM, 20× Sypro Orange, in 20 mM Tris-HCl, 150 mM NaCl, pH 7.4, and with the addition of 4 mM DDT when necessary. Unfolding was monitored over a temperature gradient from 25 to 95°C with 1°C min^−1^ ramp rate. Data were normalised and melting transitions assigned as the peak maximum of the first differential of the unfolding curve.

### Superoxide dismutase activity and free thiol assay

One hundred micromolar wild-type and mutant hCCS in 20 mM Tris-HCl, pH 7.5, 150 mM NaCl, 0.5 mM DTT was incubated with stoichiometric amounts of tetrakis(acetonitrile)copper(I) hexafuorophosphate on ice for 30 minutes. This results in 87% copper occupancy [28]. Zinc-metalated, wild-type SOD1 was reduced with 40 mM DTT overnight at 4°C and desalted into oxygen-free 50 mM potassium phosphate buffer, pH 7.4, with a Minitrap G25 column under anaerobic conditions. hCCS and SOD1 were mixed stoichometrically, diluted to 12.5 μM with oxygenated 50 mM potassium phosphate, pH 7.4, and incubated at room temperature, with samples taken at 30 and 60 minutes for activity or thiol assays. SOD1 activity was measured according to McCord and Fridovich [52]; absorbance at 550 nm was measured from a solution of 50 mM potassium phosphate, pH 7.4, 0.2 mM EDTA, 20 μM equine heart cytochrome c′, 50 μM xanthene, 0.007 units of xanthene oxidase, and 50 picomoles of SOD1-hCCS complex. Activity was calculated from ΔA_550 nm_/Δtime between time 0 and 20 seconds, with hCCS mutant activity stated as a percentage of wild-type SOD1 activity by interaction with wild-type hCCS.

hCCS-SOD1 complex-free thiols were blocked with a 20-fold excess 4-acetamido-4′-maleimidylstilbene-2,2′-disulfonic acid for 2 hours at 37°C, and then proteins were separated by reducing, denaturing 15% SDS-PAGE.

### Surface charge and dihedral analysis

Surface charge was calculated using the Coulombic Surface Coloring tool within Chimera [53] using dialectic constant 4.0, d 1.4 Å. Dihedral angles were calculated using Biopython [54]. Dihedral angle changes on SOD1 disulphide formation were calculated by averaging across two high resolution hSOD1 structures (PDB: 2C9V and 2V0A) and subtraction of the average hSOD1 angles from the compact conformer structure presented here.

### Complexation assay

Complexation between dimer interface mutants was observed as previously described [28], but over a time course from 8 minutes to 4 days, using an Agilent BioSec Advance 300 Å 4.6 × 300 mm SEC column. Complexation was then plotted as a function of the heterodimer peak height on elution from size exclusion chromatogram and rendered on a log scale to aid curve fitting. The complexation reaction does not go to completion for Ala231Gly hCCS, with some homodimeric SOD1 and hCCS remaining in solution after 4 days. Analysis of oligomeric states was performed as above or using a Superdex 75 10 × 300 mm SEC column.

### Membrane association

Lipid membranes were prepared from cholesterol (CHOL), 1-palmitoyl-2-oleoyl-sn-glycero-3-phospho-L-serine (POPS), and 1,2-dipalmitoyl-sn-glycero-3-phosphocholine (PC) at 1:7:2, respectively. CHOL, POPS, and PC powder were dissolved in chloroform and dried under nitrogen gas. To generate the liposome, 500 nmol of lipids were hydrated with 50 μL of 20 mM Tris-HCl, pH 7.4, 150 mM NaCl, and incubated with agitation at room temperature for 45 minutes. The lipid was sonicated in a water bath. Liposomes were incubated with 5 μg of protein at 37°C for 60 minutes. Reactions were centrifuged at 16,000*g* for 30 minutes at 22°C and the supernatant removed. The lipid pellet was resuspended in 200 μL buffer, pelleted, and the supernatant discarded. The liposome pellet was resuspended in 24 μL of buffer and 6 μμL of 4× SDS-PAGE sample buffer. Cytochrome bc1 complex was used as positive control in 25 mM potassium phosphate, pH 7.5, 100 mM NaCl, 3 mM sodium azide, 0.015% DDM. The supernatant and pellet fractions were analysed by reducing, denaturing SDS-PAGE, and densitometry was performed with ImageJ.

### Sequence analysis

Human SOD1 and hCCS sequences were compared with the BLAST Model Organisms Database and the OMA database. Prokaryotic sequences were removed before alignment with Clustal Omega and visualisation with WebLogo 3.0. A few eukaryotic sequences with atypical insertions in the regions of interest were also removed to aid visualisation.

## Supporting information

S1 Fig The multiple functions of hCCS and crystallographic structures presented here. **(a)** Flow diagram of CCS functional processes showing downstream outcomes of each step (green), including SOD1 folding [7,55], stability and degradation [56], peroxisomal import [57], dismutase activity [58], and mitochondrial localisation [59]. Potential negative consequences of CCS functional inefficacy at each step are highlighted (red). (**b**) hSOD1 (cyan) and hCCS SOD1-like domain 2 (pink) molecules have RMSD 0.26 and 0.25 Å, respectively. Each full-length hCCS structure represents the classic antiparallel Greek-key β-barrel dimeric assembly [60], with added functionality grafted onto the domain II SOD1-like core, with the addition of N-terminal αβ-plait, ferredoxin-fold domain I and C-terminal CXC motif domain III. Copper binding resides in a typical MXCXXC motif on the surface of domain I and hSOD1 thiol oxidation is thought to involve the atypical CXC motif in domain III [3,2]. CCS, copper chaperone for SOD1; CXC, Cys-Xxx-Cys; hCCS, human copper chaperone for SOD1; hSOD1, human superoxide dismutase-1; MXCXXC, Met-Xxx-Cys-Xxx-Xxx-Cys; RMSD, root mean square deviation; SOD1, superoxide dismutase-1.
(PNG)

S2 Fig Structure and dimer affinity of the SOD1-interacting hCCS domain II. (**a**) The two canonical SOD1 inter-subunit hydrogen bonds. (**b**) SOD1-like hydrogen binding is conserved at the hCCS domain II dimer interface despite substitution of SOD1 Ile151, which is central to intra-subunit hydrogen bonding, for the larger and charged hCCS Arg232. Arg232 side-chain guanidinium is found 4.0 Å from the similarly charged Arg196 guanidinium. (**c**) Coulombic surface charge maps indicate that this creates an electropositive dimer interface (blue, positive; red, negative). Progressive loss of repulsive dimer interface interactions in the order hCCS>hCCS-SOD1>SOD1 follows the necessary relative dimer affinities of each species. (**d**) SECs of Ala231Gly hCCS, which has an increased relative amount of dimeric species in comparison with the wild-type form. Increased absorbance in the elution tail of wild-type hCCS is indicative of more monomeric species. (**e**) Superoxide dismutase activity conferred by hCCS variants, measured by inhibition of cytochrome c′ reduction at 30 and 60 minutes following complexation between Cu-hCCS and SOD1. Data shown are the mean of three assays. The table shows relative activity of SOD1 when activated by hCCS variants, stated as a percentage of wild-type SOD1 activated by wild-type hCCS for 60 minutes, with standard error and linear regression *R*^2^ values given. (**f**) hCCS-catalysed SOD1 disulphide formation measured by AMS conjugation to free thiols and reducing SDS-PAGE. Upper gel, individual components before complexation with and without AMS. Lower gel, time course of SOD1 disulphide formation by hCCS variants. Bands representing disulphide-reduced SOD1 (red boxes) progressively diminish over 60 minutes following complexation with wild-type, R104H, and R104A hCCS, whereas A231G hCCS has limited ability to oxidise the thiols. AMS, 4-acetamido-4′-maleimidylstilbene-2,2′-disulfonic acid; hCCS, human copper chaperone for SOD1; SEC, size exclusion chromatogram; SOD1, superoxide dismutase-1.
(PNG)

S3 Fig The hCCS SOD1-like domain intra-subunit disulphide. (**a**) 2Fo-Fc electron density maps contoured at 1σ of the disulphide bond between hCCS cysteines 141 and 227 in the homodimer and heterodimer states. In each case, sulphurs are 2.1 Å apart. (**b**) Disulphide-intact and disulphide-reduced hCCS domain II can form a monodisperse heterodimer with SOD1. hCCS disulphide reduction shifts the complex elution position, indicating increased hydrodynamic radius and a looser conformation. (**c**) DSF unfolding curves for hCCS domain II in the disulphide-intact or -reduced state. Reduction of the hCCS intra-subunit disulphide bond destabilises the hCCS homodimer (disulphide-reduced and -intact T_m_ 44.1 and 59.4°C, respectively) and (**d**), the hCCS domain II complex with SOD1 (disulphide-reduced and -intact T_m_ 50.4 and 63.0°C, respectively, with a low-temperature melting transition of 48.7°C maintained in the complex state). DSF, differential scanning fluorimetry; hCCS, human copper chaperone for SOD1; SOD1, superoxide dismutase-1.
(TIF)

S4 Fig SOD1 disulphide sub-loop induced-fit conformational change. (**a**) Alignment of wtSOD1 (grey) with Cys57/146Ala SOD1 (cyan). Mutagenic removal of the SOD1 disulphide bond does not change the conformation of the overall protein in the homodimer state, 0.2 Å RMSD. (**b**) The conformation of the disulphide sub-loop, the position of residues 57 and 146, and by extension the conformation of the active site Arg143 are preserved in comparison with wild-type, disulphide-intact, dimeric SOD1 (2Fo-Fc electron density map contoured at 1σ). (**c-i)** Complexation with hCCS induces a SOD1 disulphide sub-loop conformation different from every chemically or mutagenically reduced SOD1 form previously observed. Complex conformation (cyan) is compared with alternative conformations (grey), with whole molecule RMSD stated. (**c**) Dimeric disulphide-reduced Cu-apo, Zn-holo wild-type SOD1 (solution NMR, PDB: 2AF2), RMSD 1.12 Å. (**d**) Monomeric Cu-apo, Zn-holo C6A, H46S, H48S, F50E, G51E, C111A, H120S SOD1 (crystal, PDB: 2XJL), RMSD 0.32 Å. (**e**) Monomeric Cu-holo, Zn-holo C6A, F50E, G51E, C111A 2XJK SOD1 (crystal, PDB: 2XJK), RMSD 0.3 Å. (**f**) Dimeric Cu-holo, Zn-holo C6A, C57A, C111A,C146A (crystal, PDB: 2GBV), RMSD 0.25 Å. (**g**) Monomeric Cu-apo, Zn-apo C6A, C111A, C57A, C146A SOD1 (crystal, PDB: 2GBU) RMSD 0.63 Å. (**h**) Dimeric Cu-apo, Zn-apo C57S SOD1 (crystal, PDB: 4MCN), RMSD 0.4 Å. (**i**) Dimeric as-isolated Cu,Zn C57S SOD1 (crystal, PDB: 4MCM), RMSD 0.25 Å. C57S SOD1. **(g)** and **(h)** show extensive conformational rearrangement around Gly56, together with Arg104/Arg232-DNT hydrogen bonding distances that are too long to enable complexation with hCCS. (**j**) The SOD1 induced-fit conformation is stabilised by several novel disulphide sub-loop internal hydrogen bonds. Hydrogen bonds conserved from disulphide-intact SOD1 are shown in black. New hydrogen bonds are shown in green (S2 Table). These interactions stabilise the complexation-induced SOD1 sub-loop conformation that separates disulphide bonding residues, as seen in Fig 2A. apo, metal-free; DNT, Asp52-Asn53-Thr54; hCCS, human copper chaperone for SOD1; holo, copper and zinc metalated; NMR, nuclear magnetic resonance; PDB, Protein Data Bank; RMSD, root mean square deviation; SOD1, superoxide dismutase-1; wt, wild-type.
(PNG)

S5 Fig Conservation of hCCS Arg104-GDXT motif interactions. (**a**) Eukaryotic Cu/ZnSOD disulphide sub-loop sequence conservation. The intra-subunit disulphide-bonding cysteine is shown in green and the DXT triad is highlighted purple. Glycine residues involved in disulphide sub-loop rearrangement and hydrogen bonding are shown in pink. The mechanisms of CCS-SOD1 molecular recognition and complexation are extensively conserved; however, there are some exceptions. *Candida albicans* SOD4, -5, and -6 do not have GDXT tetrads [61]. The *C*. *albicans* CCS ortholog contains Arg104, but this is more likely to facilitate interaction with SOD1, which does have a GDNT motif. SOD5 is monomeric, does not bind zinc, and cannot be activated by *Saccharomyces cerevisiae* yCCS [62]. Indeed, SOD4, -5, and -6 are extracellular enzymes and acquire copper from the surrounding environment. The GDXT motif is completely absent in prokaryotic Cu/ZnSODs, which can acquire copper from the copper chaperone CueP and auto-oxidise their disulphide in the less reducing environment of the periplasm. These SODs have an extended disulphide sub-loop and are often monomeric [63] or dimerise through a distinct interface [64]. *Caenorhabditis elegans* does not possess a CCS ortholog, but both intracellular Cu/ZnSOD homologs, SOD1 and SOD5, have intact GDXT motifs [26]. Polar amino acids on the solvent exposed surface of the disulphide sub-loop may have a solubility benefit that has favoured their retention, even without copper loading by CCS. (**b**) Copper chaperone for SOD1 domain II multiple sequence alignment. The DXT/S triad is highlighted purple and intra-subunit disulphide cysteines green, with Plantae and Fungi alternatives shown in yellow. The highly conserved Arg104 (human numbering) is shown in orange. Perhaps the most obvious exception to the R104-DXT rule is SOD1 itself. Asparagine-19 is found at the midpoint of human SOD1 β-strand 2. The 7-Å side-chain distances negate the possibility of an Asn19-DNT interaction. As a result, when the SOD1 disulphide is in the reduced state, there can be no compensatory stabilisation across the dimer interface. (**c**) Mutation of hCCS Arg104 thermally destabilises the hCCS homodimer. DSF unfolding of wild-type hCCS; Arg104Ala hCCS, which completely removes the interaction with the SOD1 GDNT motif; and Arg104His hCCS, which maintains the charge but increases the noncovalent interaction distances. Single unfolding transitions are observed for wild-type hCCS (60.2°C), while two unfolding transitions are observed for both Arg104 mutations: R104A hCCS 50.3 and 57.2°C, R104A hCCS-SOD1 51.3 and 59.2°C. (**d**) Alignment of human, cetacean, and other relevant CCS protein sequences. Human Arg104 is highlighted green and the cetacean histidine substitution is in blue. Of the baleen whales and toothed whales, where sequence data are available, all have histidine at this site. The R104 mutation is not observed in other clades or suborders within the Artiodactyla or other groups of marine mammals. The histidine substitution must have occurred before diversification of the two whale parvorders Mysticeti and Odontoceti, roughly 30 mya. The reasons for the subsequent conservation of this substitution are obscure. Histidine maintains the charge but shortens the amino acid side chain and, by necessity, must strain the His-DXT interaction by extending the bonding distance. (**e**) DSF unfolding of wild-type hCCS-SOD1 (63.1°C); R104H hCCS (53.2 and 61.2°C); R104H hCCS-SOD1 (53.2 and 63.2°C). Disulphide-reduced, wild-type SOD1 has a single unfolding transition at 54.2°C (orange). CCS, copper chaperone for SOD1; Cu/ZnSODs, copper/zinc superoxide dismutases; DNT, Asp52-Asn53-Thr54; DSF, differential scanning fluorimetry; DXT, Asp-Xxx-Thr; DXT/S, Asp-Xxx-Thr/Ser; GDNT, Gly51-Asp52-Asn53-Thr54; GDXT, Gly51-Asp52-Xxx-Thr54; hCCS, human copper chaperone for SOD1; mya, million years ago; yCCS, yeast copper chaperone for SOD1.
(PNG)

S6 Fig Conformational plasticity of the hCCS-SOD1 complex facilitates SOD1 activation. (**a**) Mobility of the hCCS copper-binding domain I. When domain I occupies a conformation close to the connected domain II (pink), interactions secure the association: Glu116 side-chain carboxylate forms four hydrogen bonds with Ser59, and Arg182 guanidinium hydrogen bonds with the Thr10 carbonyl. These interactions are broken to affect a domain swap (orange). In this state, the Glu116-Ser59 interaction is weakened and the domain I orientation is not identical. (**b**) Lattice arrangement within the compact conformer crystal creates clashes (red) that prevent one hCCS domain I from forming contacts that stabilise it close to domain II. As a result, electron density for this hCCS monomer begins at residue Asn85, within the domain I–domain II linker, and ends at Leu236. (**c**) hCCS domain III amino acids 241–246 and 250–255 form the antiparallel β-sheet interface of an octameric supramolecular assembly in crystallo. This β-sheet and the position of the C-terminal cysteines within it are conserved in a yCCS-hSOD1 chimeric structure [28] (PDB: 5U9M), bringing cysteines involved in SOD1 disulphide formation into close proximity—here, hCCS Cys246, and in yCCS, Cys231-ySOD1 Cys146. Changing the position and spacing of the C-terminal cysteines is severely detrimental to SOD1 activation and disulphide formation [12]. (**d**) hCCS domain I can stabilise domain III in a position interacting with the SOD1 disulphide loop. (**e**) Hydrogen bonding interactions within domain III and between domain III and domain I or SOD1. Amino acids 235–241 are positioned to bring functional residues 244 and 246 close to SOD1 Cys57. This conformation therefore facilitates formation of the disulphide-linked complex described previously for the yeast orthologue [33]. In contrast, the yCCS-hSOD1 chimera structure shows domain I significantly shifted from its position in comparison with those of nonchimeric heterodimer forms [12]. This is due to accommodation of tetrahedral zinc coordination by the domain I copper binding cysteines in two symmetry-related molecules. (**f**) Formation of the SOD1 disulphide bond shifts the position of the disulphide sub-loop and breaks hydrogen bonding between hCCS Asn239 and the SOD1 Thr58 (2.7 to 6.7 Å). Blue, hCCS domain III; cyan, SOD1 disulphide loop within the complex; pink, SOD1 disulphide loop conformation after formation of the SOD1 disulphide (2C9V). hCCS, human copper chaperone for SOD1; hSOD1, human superoxide dismutase-1; PDB, Protein Data Bank; SOD1, superoxide dismutase-1; yCCS, yeast copper chaperone for SOD1.
(PNG)

S7 Fig Structural basis of hCCS membrane association. (**a**) Sequence alignment of yeast and human CCS proteins showing the residues known to facilitate yCCS association with membranes (purple) [9]. The charge of these residues are not conserved (with the exception of Arg172) in the human sequence or the structure of β-barrel loops I, IV, and VII, which are shortened or conformationally rearranged to form a highly electronegative β-barrel end surface. It is noteworthy that changes to the sequence and position of loops I and VII (Fig 3A) are a direct result of hCCS domain II zinc binding, a PTM not present in yCCS. There appears to have been an evolutionary divergence in the structure and behaviour of CCS orthologues from the Animalia kingdom that bind zinc, have an internal disulphide bond and relatively high dimer affinity, and associate with membranes through the association of domain I and II, compared with yCCS, which has low dimer affinity, does not bind zinc, and membrane associates through positively charged residues on the β-barrel surface of the domain II end [9,27,65]. (**b**) The copper chaperone protein Atox1 has 28% sequence identity with hCCS domain I, interacts with Ctr1, and associates with membranes [66]. Sequence alignment of human Atox1 and hCCS domain I, showing lack of conservation of positively charged residues known to mediate Atox1–lipid membrane association (purple). However, Arg30 and Lys31 (blue), which are proximal to the domain I copper binding site, do facilitate membrane association (Fig 5C). (**c**) SEC and light scattering–derived molecular weights showing that hCCS domain I–II is monomeric at mid–low micromolar concentration. (**d**) SEC and light scattering–derived molecular weights showing that hCCS domain II–III is dimeric. Masses are quoted as experimental/predicted. Each species has a polydispersity index of 1.0. (**e**) Homodimeric structure of full-length hCCS, with surface coloured by Coulombic charge showing the positively charged region Arg225 to Lys267 within domain III. This region evidently does not provide enough surface area to mediate membrane association. Homodimeric hCCS was generated by alignment of compact hCCS conformers to the hCCS domain II structure and refinement of domain I and III positions against SAXS data using the method described previously [28,35], which (**f**) fits the experimental data with a χ value of 1.51. CCS, copper chaperone for SOD1; Ctr1, copper transporter-1; hCCS, human copper chaperone for SOD1; PTM, posttranslation modification; SAXS, small-angle x-ray scattering; SEC, size-exclusion chromatography; yCCS, yeast copper chaperone for SOD1.
(PNG)

S1 Table Crystallographic data collection and refinement statistics. (DOCX)

S2 Table SOD1 interface hydrogen bonding interactions. SOD1, superoxide dismutase-1.
(DOCX)

S3 Table hCCS domain II interface noncovalent bonding interactions. hCCS, human copper chaperone for SOD1
(DOCX)

S4 Table hCCS-SOD1 complex interface noncovalent bonding interactions. hCCS, human copper chaperone for SOD1; SOD1, superoxide dismutase-1.
(DOCX)

S5 Table Internal SOD1 disulphide sub-loop hydrogen bonding in the disulphide-intact homodimeric state in comparison with the conformation present in the heterodimeric complex with hCCS. hCCS, human copper chaperone for SOD1; SOD1, superoxide dismutase-1.
(DOCX)

S6 Table The effect of SOD1 disulphide bond formation on hCCS-SOD1 inter-subunit noncovalent bonding interactions. hCCS, human copper chaperone for SOD1; SOD1, superoxide dismutase-1.
(DOCX)

S1 Data Underlying data for the following Figs 2D, 5C, S2D, S2E, S3, S5C, S5E, S7C, S7D and S7F. (XLSX)

## Abbreviations

ALS: amyotrophic lateral sclerosis
ASU: asymmetric unit
CCS: copper chaperone for SOD1
CHOL: cholesterol
Ctr1: copper transporter-1
Cu/ZnSOD: copper/zinc superoxide dismutase
CXC: Cys-Xxx-Cys
DTT: dithiothreitol
GDNT: Gly51-Asp52-Asn53-Thr54
GDXT: Gly51-Asp52-Xxx-Thr54
hCCS: human copper chaperone for SOD1
hSOD1: human superoxide dismutase-1
NMR: nuclear magnetic resonance
PC: 1,2-dipalmitoyl-sn-glycero-3-phosphocholine
POPS: 1-palmitoyl-2-oleoyl-sn-glycero-3-phospho-L-serine
PTM: posttranslation modification
SAXS: small-angle x-ray scattering
SOD1: superoxide dismutase-1
Tris-HCl: *tris*(hydroxymethyl)aminomethane-HCl
yCCS: yeast copper chaperone for SOD1
