# Supplementary figures and images for: Molecular recognition and maturation of SOD1 by its evolutionarily destabilised cognate chaperone hCCS
Source: PLoS Biol. 2019 Feb 8;17(2):e3000141. doi: 10.1371/journal.pbio.3000141 (PMC6383938; doi:10.1371/journal.pbio.3000141)

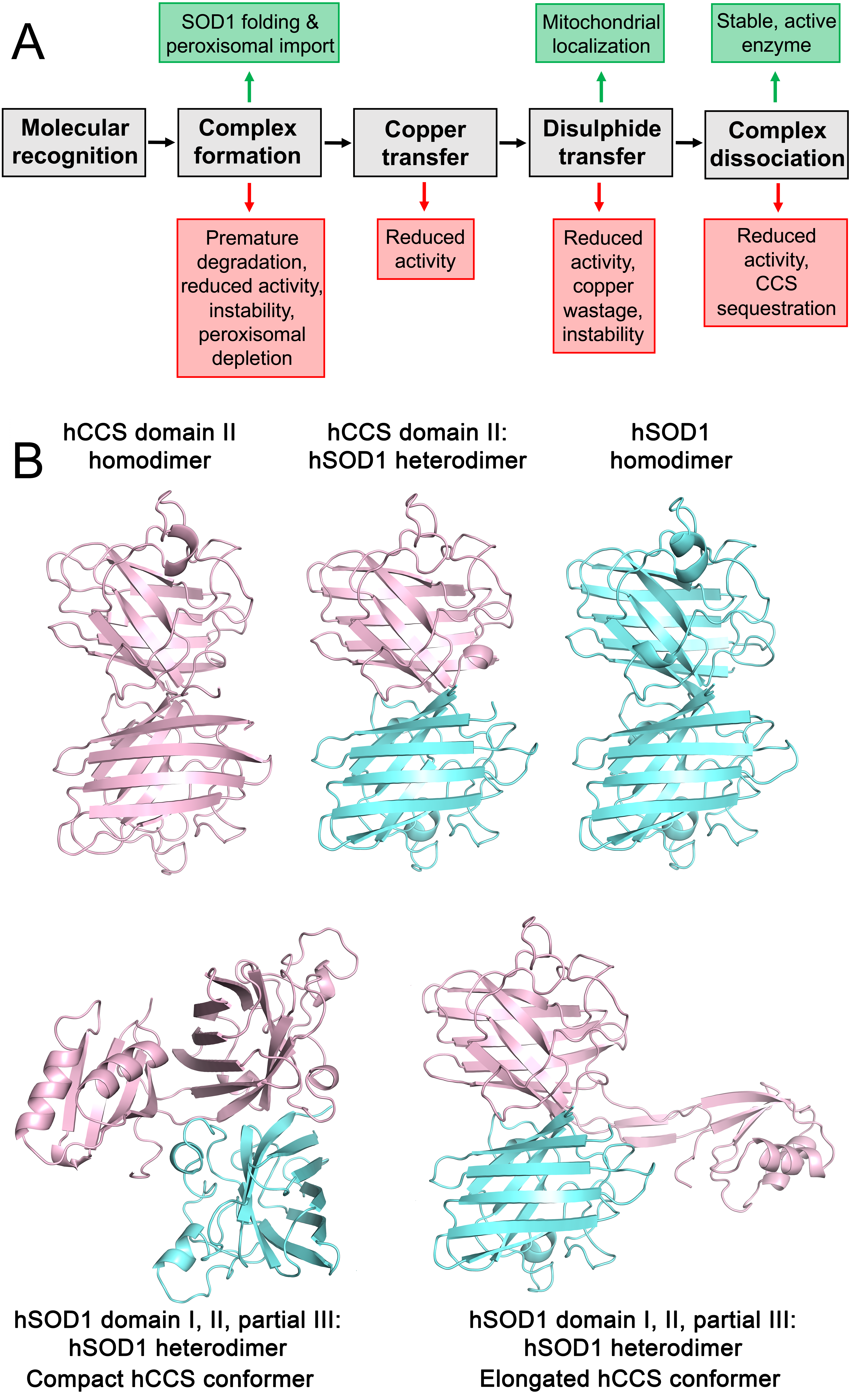

Supplement: S1 Fig — (a) Flow diagram of CCS functional processes showing downstream outcomes of each step (green), including SOD1 folding [7,55], stability and degradation [56], peroxisomal import [57], dismutase activity [58], and mitochondrial localisation [59]. Potential negative consequences of CCS functional inefficacy at each step are highlighted (red). (b) hSOD1 (cyan) and hCCS SOD1-like domain 2 (pink) molecules have RMSD 0.26 and 0.25 Å, respectively. Each full-length hCCS structure represents the classic antiparallel Greek-key β-barrel dimeric assembly [60], with added functionality grafted onto the domain II SOD1-like core, with the addition of N-terminal αβ-plait, ferredoxin-fold domain I and C-terminal CXC motif domain III. Copper binding resides in a typical MXCXXC motif on the surface of domain I and hSOD1 thiol oxidation is thought to involve the atypical CXC motif in domain III [3,2]. CCS, copper chaperone for SOD1; CXC, Cys-Xxx-Cys; hCCS, human copper chaperone for SOD1; hSOD1, human superoxide dismutase-1; MXCXXC, Met-Xxx-Cys-Xxx-Xxx-Cys; RMSD, root mean square deviation; SOD1, superoxide dismutase-1. (PNG) [file pbio.3000141.s001.png]

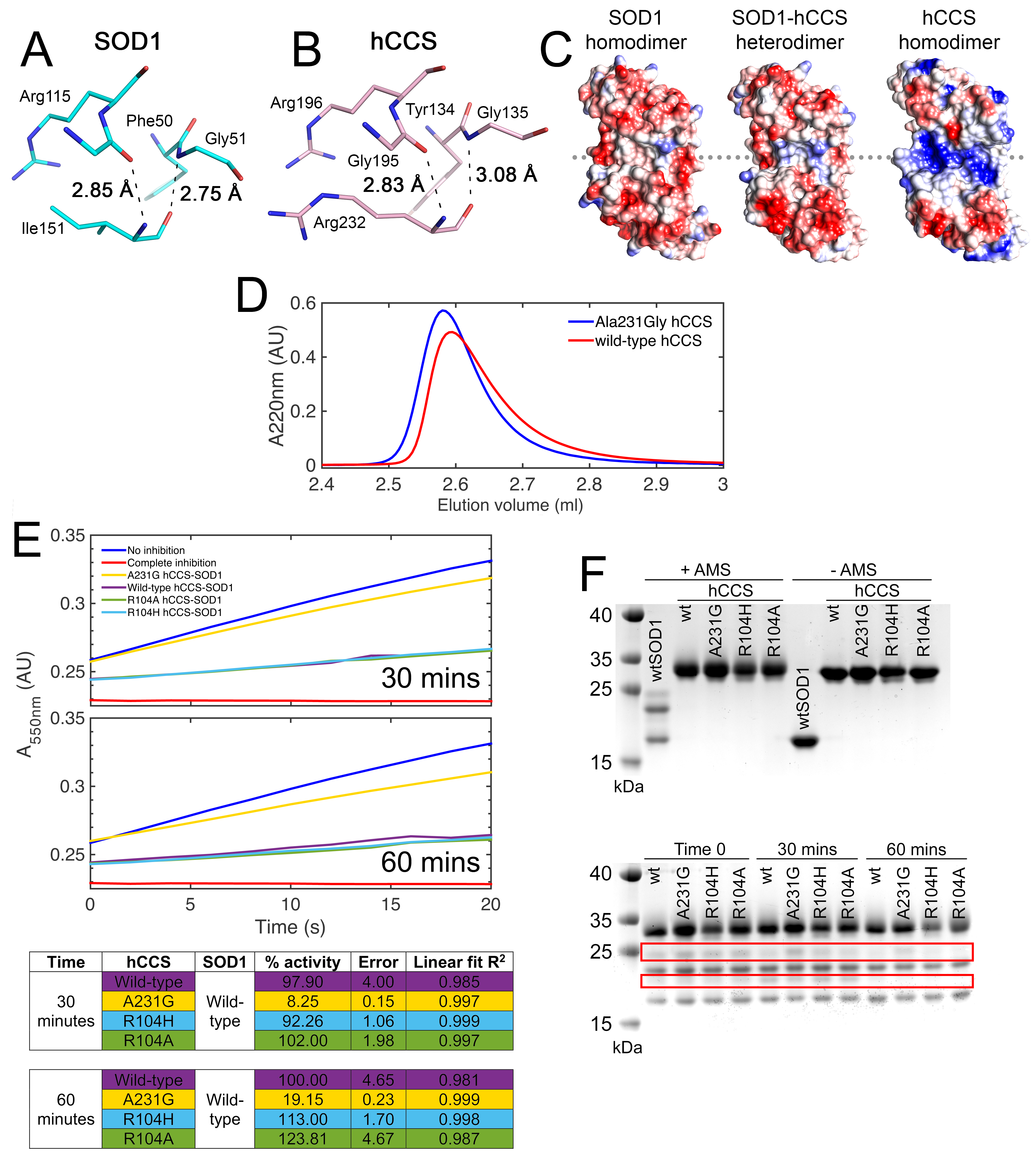

Supplement: S2 Fig — (a) The two canonical SOD1 inter-subunit hydrogen bonds. (b) SOD1-like hydrogen binding is conserved at the hCCS domain II dimer interface despite substitution of SOD1 Ile151, which is central to intra-subunit hydrogen bonding, for the larger and charged hCCS Arg232. Arg232 side-chain guanidinium is found 4.0 Å from the similarly charged Arg196 guanidinium. (c) Coulombic surface charge maps indicate that this creates an electropositive dimer interface (blue, positive; red, negative). Progressive loss of repulsive dimer interface interactions in the order hCCS>hCCS-SOD1>SOD1 follows the necessary relative dimer affinities of each species. (d) SECs of Ala231Gly hCCS, which has an increased relative amount of dimeric species in comparison with the wild-type form. Increased absorbance in the elution tail of wild-type hCCS is indicative of more monomeric species. (e) Superoxide dismutase activity conferred by hCCS variants, measured by inhibition of cytochrome c′ reduction at 30 and 60 minutes following complexation between Cu-hCCS and SOD1. Data shown are the mean of three assays. The table shows relative activity of SOD1 when activated by hCCS variants, stated as a percentage of wild-type SOD1 activated by wild-type hCCS for 60 minutes, with standard error and linear regression R2 values given. (f) hCCS-catalysed SOD1 disulphide formation measured by AMS conjugation to free thiols and reducing SDS-PAGE. Upper gel, individual components before complexation with and without AMS. Lower gel, time course of SOD1 disulphide formation by hCCS variants. Bands representing disulphide-reduced SOD1 (red boxes) progressively diminish over 60 minutes following complexation with wild-type, R104H, and R104A hCCS, whereas A231G hCCS has limited ability to oxidise the thiols. AMS, 4-acetamido-4′-maleimidylstilbene-2,2′-disulfonic acid; hCCS, human copper chaperone for SOD1; SEC, size exclusion chromatogram; SOD1, superoxide dismutase-1. (PNG) [file pbio.3000141.s002.png]

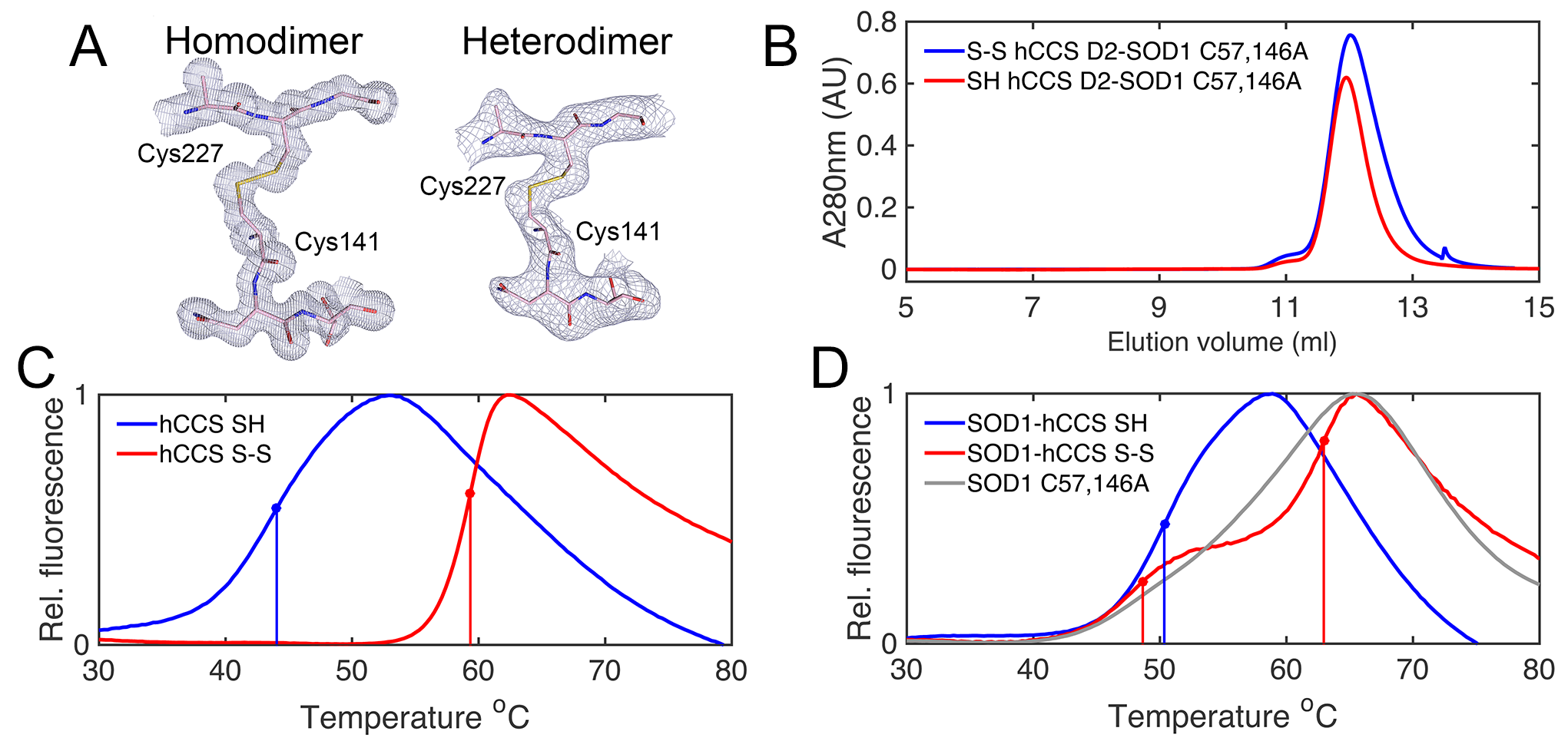

Supplement: S3 Fig — (a) 2Fo-Fc electron density maps contoured at 1σ of the disulphide bond between hCCS cysteines 141 and 227 in the homodimer and heterodimer states. In each case, sulphurs are 2.1 Å apart. (b) Disulphide-intact and disulphide-reduced hCCS domain II can form a monodisperse heterodimer with SOD1. hCCS disulphide reduction shifts the complex elution position, indicating increased hydrodynamic radius and a looser conformation. (c) DSF unfolding curves for hCCS domain II in the disulphide-intact or -reduced state. Reduction of the hCCS intra-subunit disulphide bond destabilises the hCCS homodimer (disulphide-reduced and -intact Tm 44.1 and 59.4°C, respectively) and (d), the hCCS domain II complex with SOD1 (disulphide-reduced and -intact Tm 50.4 and 63.0°C, respectively, with a low-temperature melting transition of 48.7°C maintained in the complex state). DSF, differential scanning fluorimetry; hCCS, human copper chaperone for SOD1; SOD1, superoxide dismutase-1. (TIF) [file pbio.3000141.s003.tif]

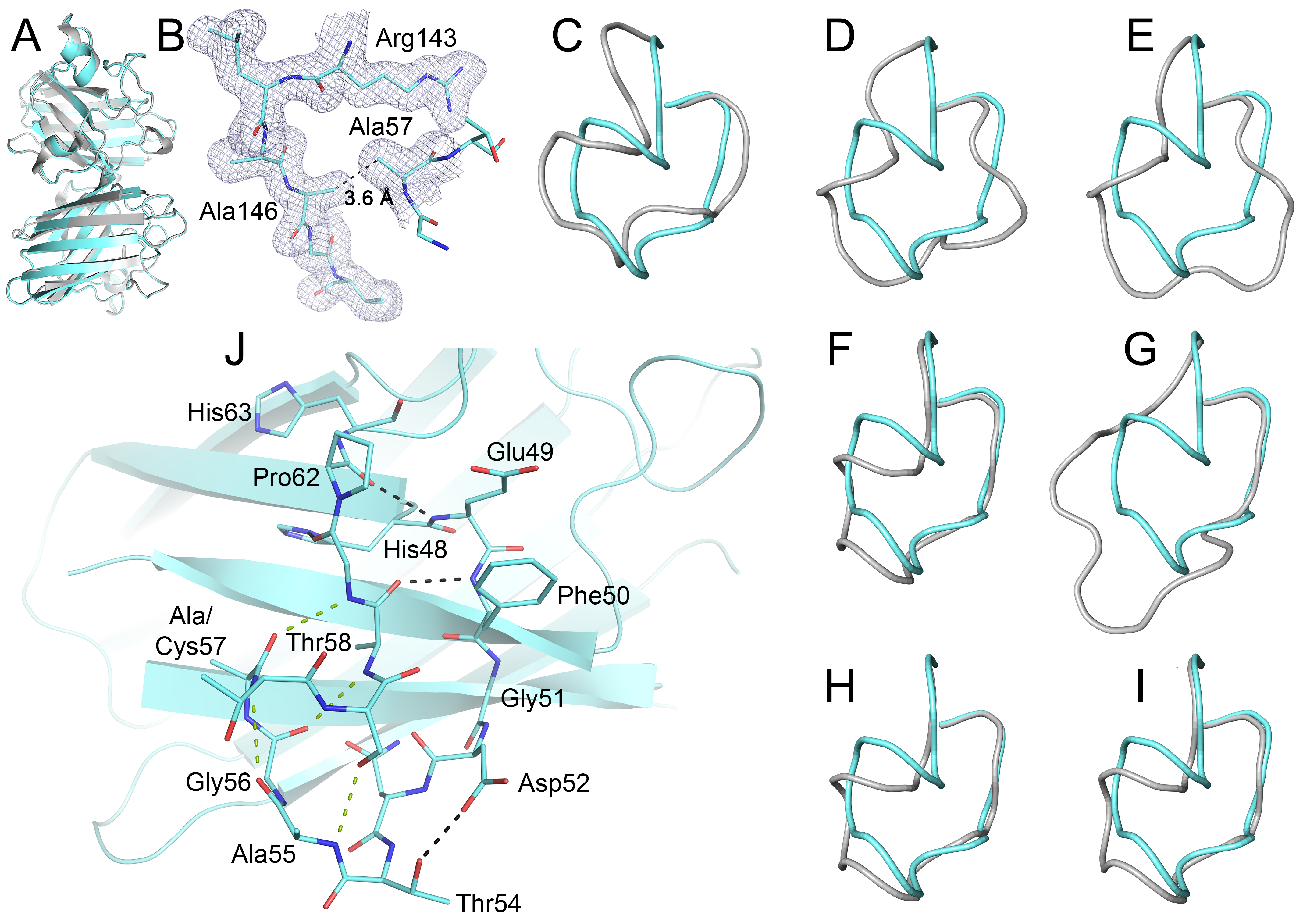

Supplement: S4 Fig — (a) Alignment of wtSOD1 (grey) with Cys57/146Ala SOD1 (cyan). Mutagenic removal of the SOD1 disulphide bond does not change the conformation of the overall protein in the homodimer state, 0.2 Å RMSD. (b) The conformation of the disulphide sub-loop, the position of residues 57 and 146, and by extension the conformation of the active site Arg143 are preserved in comparison with wild-type, disulphide-intact, dimeric SOD1 (2Fo-Fc electron density map contoured at 1σ). (c-i) Complexation with hCCS induces a SOD1 disulphide sub-loop conformation different from every chemically or mutagenically reduced SOD1 form previously observed. Complex conformation (cyan) is compared with alternative conformations (grey), with whole molecule RMSD stated. (c) Dimeric disulphide-reduced Cu-apo, Zn-holo wild-type SOD1 (solution NMR, PDB: 2AF2), RMSD 1.12 Å. (d) Monomeric Cu-apo, Zn-holo C6A, H46S, H48S, F50E, G51E, C111A, H120S SOD1 (crystal, PDB: 2XJL), RMSD 0.32 Å. (e) Monomeric Cu-holo, Zn-holo C6A, F50E, G51E, C111A 2XJK SOD1 (crystal, PDB: 2XJK), RMSD 0.3 Å. (f) Dimeric Cu-holo, Zn-holo C6A, C57A, C111A,C146A (crystal, PDB: 2GBV), RMSD 0.25 Å. (g) Monomeric Cu-apo, Zn-apo C6A, C111A, C57A, C146A SOD1 (crystal, PDB: 2GBU) RMSD 0.63 Å. (h) Dimeric Cu-apo, Zn-apo C57S SOD1 (crystal, PDB: 4MCN), RMSD 0.4 Å. (i) Dimeric as-isolated Cu,Zn C57S SOD1 (crystal, PDB: 4MCM), RMSD 0.25 Å. C57S SOD1. (g) and (h) show extensive conformational rearrangement around Gly56, together with Arg104/Arg232-DNT hydrogen bonding distances that are too long to enable complexation with hCCS. (j) The SOD1 induced-fit conformation is stabilised by several novel disulphide sub-loop internal hydrogen bonds. Hydrogen bonds conserved from disulphide-intact SOD1 are shown in black. New hydrogen bonds are shown in green (S2 Table). These interactions stabilise the complexation-induced SOD1 sub-loop conformation that separates disulphide bonding residues, as seen in Fig 2A. apo, metal-free; DNT, Asp52-Asn53-Thr54; hCC [file pbio.3000141.s004.png]

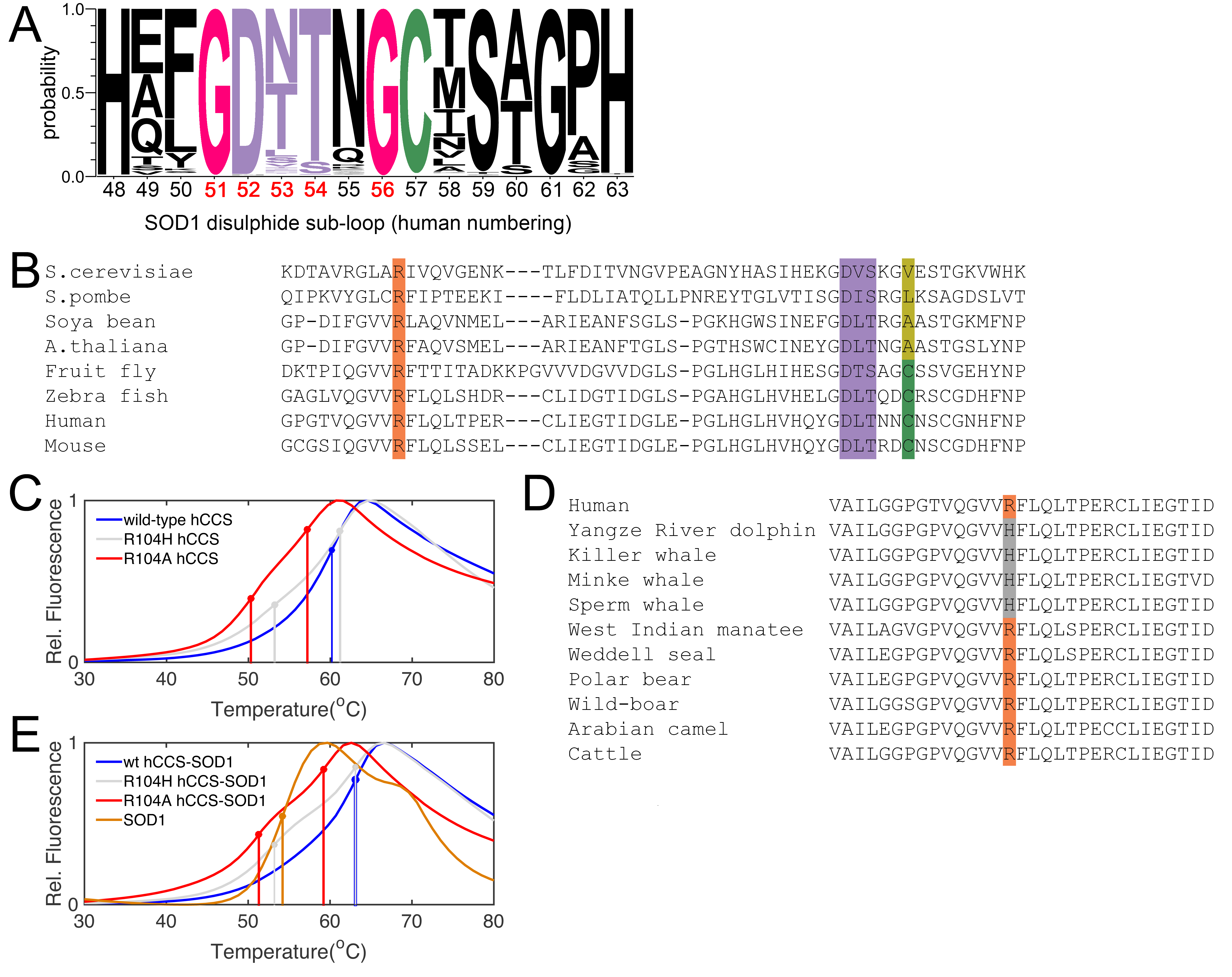

Supplement: S5 Fig — (a) Eukaryotic Cu/ZnSOD disulphide sub-loop sequence conservation. The intra-subunit disulphide-bonding cysteine is shown in green and the DXT triad is highlighted purple. Glycine residues involved in disulphide sub-loop rearrangement and hydrogen bonding are shown in pink. The mechanisms of CCS-SOD1 molecular recognition and complexation are extensively conserved; however, there are some exceptions. Candida albicans SOD4, -5, and -6 do not have GDXT tetrads [61]. The C. albicans CCS ortholog contains Arg104, but this is more likely to facilitate interaction with SOD1, which does have a GDNT motif. SOD5 is monomeric, does not bind zinc, and cannot be activated by Saccharomyces cerevisiae yCCS [62]. Indeed, SOD4, -5, and -6 are extracellular enzymes and acquire copper from the surrounding environment. The GDXT motif is completely absent in prokaryotic Cu/ZnSODs, which can acquire copper from the copper chaperone CueP and auto-oxidise their disulphide in the less reducing environment of the periplasm. These SODs have an extended disulphide sub-loop and are often monomeric [63] or dimerise through a distinct interface [64]. Caenorhabditis elegans does not possess a CCS ortholog, but both intracellular Cu/ZnSOD homologs, SOD1 and SOD5, have intact GDXT motifs [26]. Polar amino acids on the solvent exposed surface of the disulphide sub-loop may have a solubility benefit that has favoured their retention, even without copper loading by CCS. (b) Copper chaperone for SOD1 domain II multiple sequence alignment. The DXT/S triad is highlighted purple and intra-subunit disulphide cysteines green, with Plantae and Fungi alternatives shown in yellow. The highly conserved Arg104 (human numbering) is shown in orange. Perhaps the most obvious exception to the R104-DXT rule is SOD1 itself. Asparagine-19 is found at the midpoint of human SOD1 β-strand 2. The 7-Å side-chain distances negate the possibility of an Asn19-DNT interaction. As a result, when the SOD1 disulphide is in the red [file pbio.3000141.s005.png]

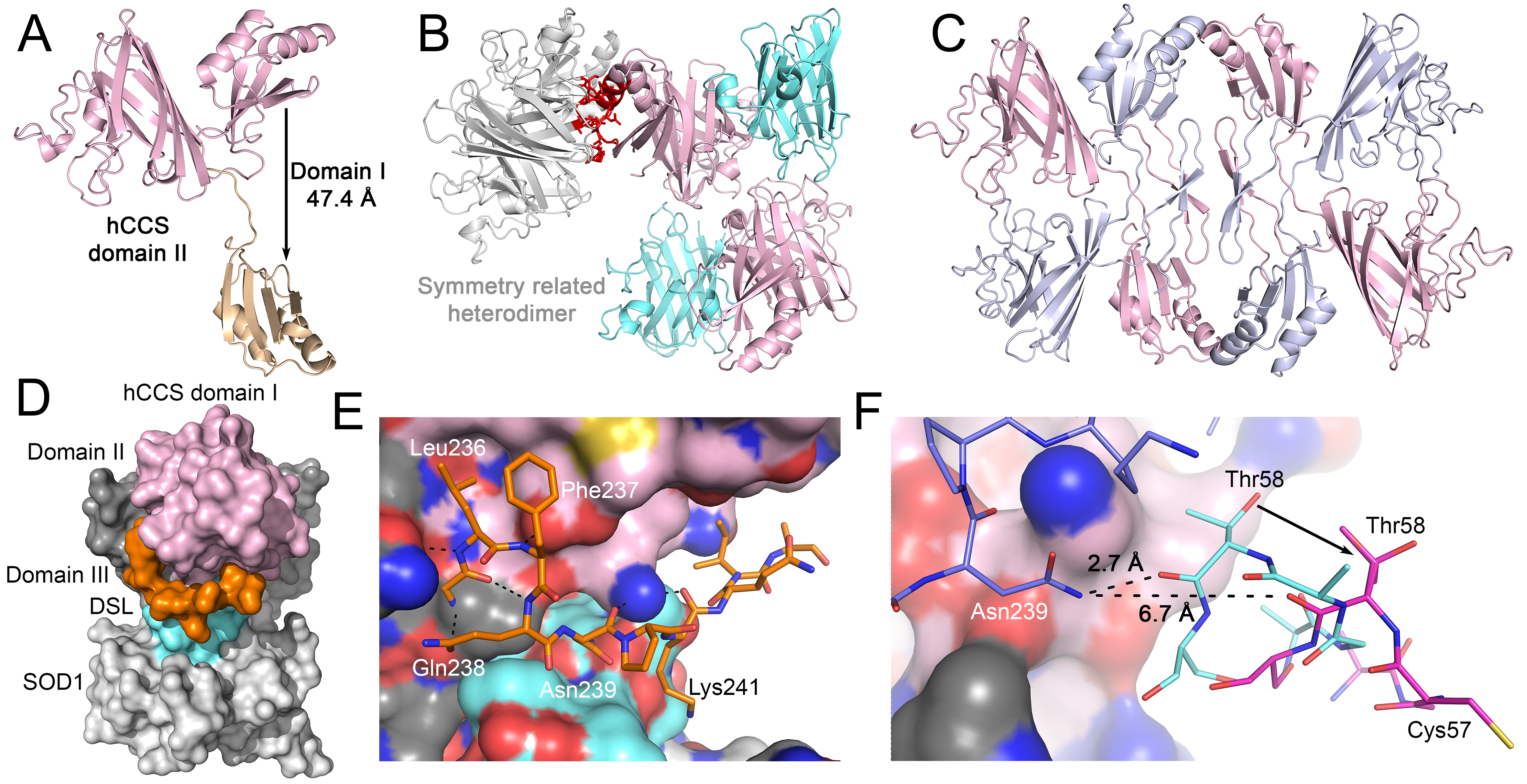

Supplement: S6 Fig — (a) Mobility of the hCCS copper-binding domain I. When domain I occupies a conformation close to the connected domain II (pink), interactions secure the association: Glu116 side-chain carboxylate forms four hydrogen bonds with Ser59, and Arg182 guanidinium hydrogen bonds with the Thr10 carbonyl. These interactions are broken to affect a domain swap (orange). In this state, the Glu116-Ser59 interaction is weakened and the domain I orientation is not identical. (b) Lattice arrangement within the compact conformer crystal creates clashes (red) that prevent one hCCS domain I from forming contacts that stabilise it close to domain II. As a result, electron density for this hCCS monomer begins at residue Asn85, within the domain I–domain II linker, and ends at Leu236. (c) hCCS domain III amino acids 241–246 and 250–255 form the antiparallel β-sheet interface of an octameric supramolecular assembly in crystallo. This β-sheet and the position of the C-terminal cysteines within it are conserved in a yCCS-hSOD1 chimeric structure [28] (PDB: 5U9M), bringing cysteines involved in SOD1 disulphide formation into close proximity—here, hCCS Cys246, and in yCCS, Cys231-ySOD1 Cys146. Changing the position and spacing of the C-terminal cysteines is severely detrimental to SOD1 activation and disulphide formation [12]. (d) hCCS domain I can stabilise domain III in a position interacting with the SOD1 disulphide loop. (e) Hydrogen bonding interactions within domain III and between domain III and domain I or SOD1. Amino acids 235–241 are positioned to bring functional residues 244 and 246 close to SOD1 Cys57. This conformation therefore facilitates formation of the disulphide-linked complex described previously for the yeast orthologue [33]. In contrast, the yCCS-hSOD1 chimera structure shows domain I significantly shifted from its position in comparison with those of nonchimeric heterodimer forms [12]. This is due to accommodation of tetrahedral zinc coordination by the domain I copper [file pbio.3000141.s006.png]

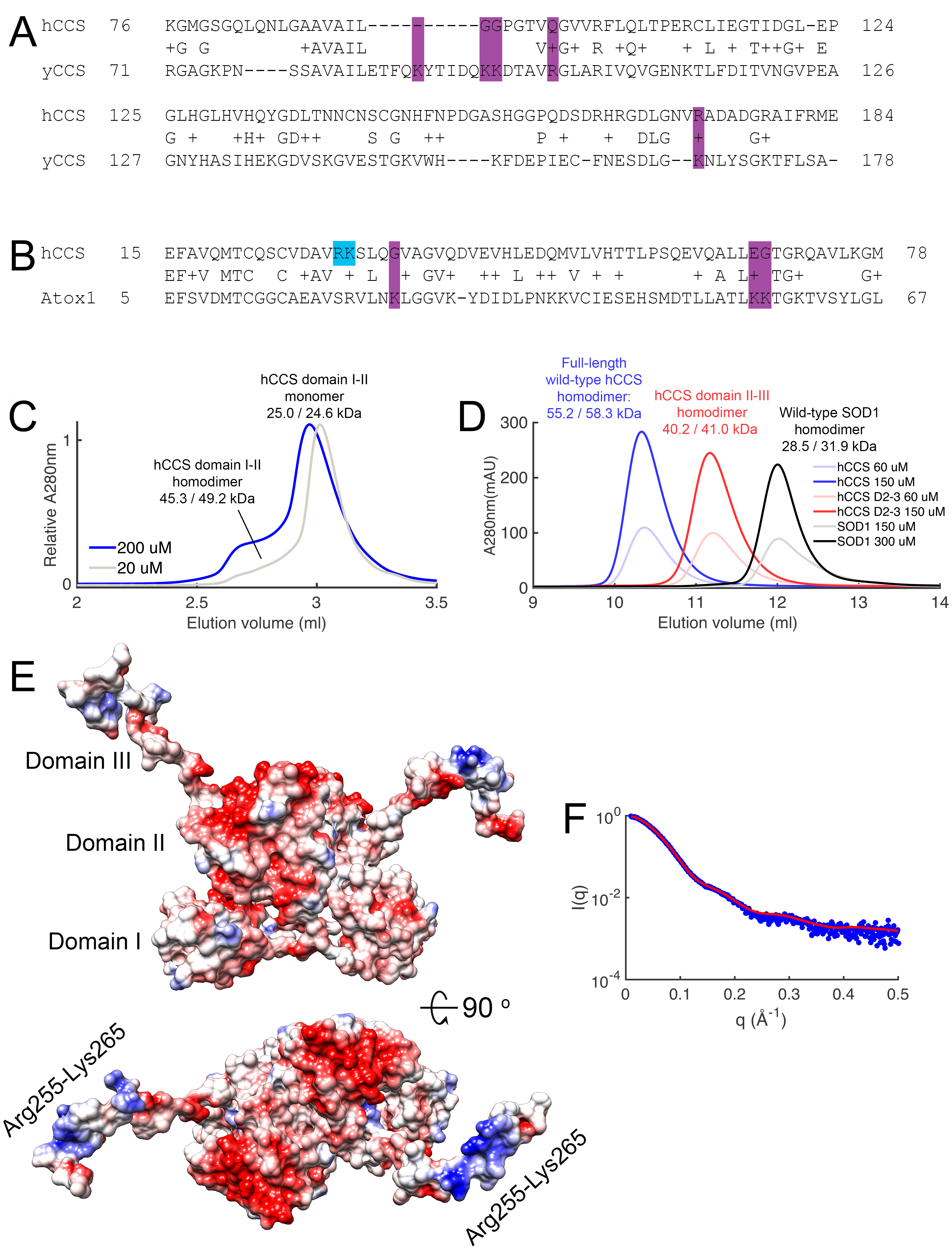

Supplement: S7 Fig — (a) Sequence alignment of yeast and human CCS proteins showing the residues known to facilitate yCCS association with membranes (purple) [9]. The charge of these residues are not conserved (with the exception of Arg172) in the human sequence or the structure of β-barrel loops I, IV, and VII, which are shortened or conformationally rearranged to form a highly electronegative β-barrel end surface. It is noteworthy that changes to the sequence and position of loops I and VII (Fig 3A) are a direct result of hCCS domain II zinc binding, a PTM not present in yCCS. There appears to have been an evolutionary divergence in the structure and behaviour of CCS orthologues from the Animalia kingdom that bind zinc, have an internal disulphide bond and relatively high dimer affinity, and associate with membranes through the association of domain I and II, compared with yCCS, which has low dimer affinity, does not bind zinc, and membrane associates through positively charged residues on the β-barrel surface of the domain II end [9,27,65]. (b) The copper chaperone protein Atox1 has 28% sequence identity with hCCS domain I, interacts with Ctr1, and associates with membranes [66]. Sequence alignment of human Atox1 and hCCS domain I, showing lack of conservation of positively charged residues known to mediate Atox1–lipid membrane association (purple). However, Arg30 and Lys31 (blue), which are proximal to the domain I copper binding site, do facilitate membrane association (Fig 5C). (c) SEC and light scattering–derived molecular weights showing that hCCS domain I–II is monomeric at mid–low micromolar concentration. (d) SEC and light scattering–derived molecular weights showing that hCCS domain II–III is dimeric. Masses are quoted as experimental/predicted. Each species has a polydispersity index of 1.0. (e) Homodimeric structure of full-length hCCS, with surface coloured by Coulombic charge showing the positively charged region Arg225 to Lys267 within domain III. This region evidently [file pbio.3000141.s007.png]
