# Supplementary material for: Molecular recognition and maturation of SOD1 by its evolutionarily destabilised cognate chaperone hCCS
Source: PLoS Biol. 2019 Feb 8;17(2):e3000141. doi: 10.1371/journal.pbio.3000141 (PMC6383938; doi:10.1371/journal.pbio.3000141)
Supplement: S1 Table — (DOCX) [file pbio.3000141.s008.docx]

**S1 Table. Crystallographic data collection and refinement statistics.**

|  | **SOD1 C57/146A homodimer^#^** | **hCCS domain II homodimer^#^** | **hCCS domain II - SOD1 C57/146A heterodimer^#^**  **(Truncated)** | **hCCS C22,25S -SOD1 C57/146A heterodimer^#^**  **(Elongated)** | **hCCS C12,22,25,244,**  **246A - SOD1 C57/146A heterodimer^#^**  **(Compact)** |
| --- | --- | --- | --- | --- | --- |
| **Data collection** |  |  |  |  |  |
| Space group | P2_1_ 2_1_ 2_1_ | P 4_3_ 2_1_ 2 | P3_2_ | P3_2_ | P3_2_ |
| **Cell dimensions** |  |  |  |  |  |
| *a*, *b*, *c* (Å) | 74.7,  163.0,  174.6 | 68.4,  68.4,  136.2 | 244.7,  244.7,  182.3 | 172.5,  172.5,  219.2 | 181.1,  181.1,  141.1 |
| α, β, γ (°) | 90, 90, 90 | 90, 90, 90 | 90, 90, 120 | 90, 90, 120 | 90, 90, 120 |
| Resolution (Å)* | 81.48-2.00  (2.03-2.00) | 68.36-1.55  (1.63-1.55) | 49.40-2.55  (2.69-2.55) | 44.19-3.05  (3.21-3.05) | 49.01-2.94  (3.10-2.94) |
| No. reflections | 144348 | 47679 | 67927 | 24104 | 110021 |
| *R*_merge_* | 8.4 (83.2) | 11.1 (83.2) | 13.0 (80.6) | 13.0 (142.9) | 16.5 (94.4) |
| *I* / σ*I** | 12.3 (1.9) | 18.8 (1.5) | 9.5 (2.0) | 18.7 (1.9) | 8.5 (1.9) |
| CC1/2 | 0.998 (0.783) | 0.998 (0.33) | 0.995 (0.409) | 0.999 (0.693) | 0.989 (0.717) |
| Completeness (%)* | 100 (100) | 100.0 (99.9) | 95.7 (97.2) | 100 (100) | 100 (100) |
| Redundancy* | 6.1 (5.7) | 105 (4.5) | 6.8 (6.7) | 14.1 (14.5) | 5.9 (5.8) |
| Wilson B-factor (Å^-2^) | 27.7 | 11.7 | 39.2 | 82.0 | 54.0 |
|  |  |  |  |  |  |
| **Refinement** |  |  |  |  |  |
| ASU content | 6 hSOD  homodimers | 1 hCCS  homodimer | 4 SOD-hCCS  heterodimers | 2 SOD-hCCS  heterodimers | 12 SOD-hCCS  heterodimers |
| Resolution (Å) | 81.48-2.00 | 61.1-1.55 | 49.40-2.55 | 42.07-3.05 | 49.01-3.0 |
| No. reflections | 136979 | 45353 | 64691 | 22855 | 98527 |
| *R*_work_ / *R*_free_ (%) | 22.1 / 26.0 | 18.6 / 21.7 | 20.8 / 24.8 | 19.9 / 24.6 | 19.2 / 22.9 |
| **No. of atoms** |  |  |  |  |  |
| Protein | 13294 | 2362 | 8925 | 5967 | 33112 |
| Water | 1064 | 393 | 270 | 2 | 75 |
| No. of residues | 1835 | 303 | 1206 | 808 | 4549 |
| **Average *B*-factors (Å^2^)** |  |  |  |  |  |
| Protein | 46.0 | 17.8 | 55.0 | 95.3 | 74.6 |
| Water | 43.8 | 33.7 | 41.4 | 78.3 | 41.9 |
| **R.M.S. deviations** |  |  |  |  |  |
| Bond lengths (Å) | 0.007 | 0.005 | 0.003 | 0.010 | 0.007 |
| Bond angles (°) | 1.15 | 0.94 | 1.67 | 1.46 | 1.15 |
|  |  |  |  |  |  |
| **PDB ID** | **6FOI** | **6FN8** | **6FOL** | **6FON** | **6FP6** |

^#^ Each data set is taken from a single crystal.

* Values in parenthesis refer to the highest resolution shell.
