# Supplementary material for: Molecular recognition and maturation of SOD1 by its evolutionarily destabilised cognate chaperone hCCS
Source: PLoS Biol. 2019 Feb 8;17(2):e3000141. doi: 10.1371/journal.pbio.3000141 (PMC6383938; doi:10.1371/journal.pbio.3000141)
Supplement: S2 Table — SOD1, superoxide dismutase-1. (DOCX) [file pbio.3000141.s009.docx]

**S2 Table. SOD1 interface hydrogen bonding interactions.**

| **Amino Acid** | **Group** | **Distance** | **Group** | **Amino Acid** |
| --- | --- | --- | --- | --- |
| Gly51 | Amine (N) | 2.75 Å* | Carbonyl (O) | Ile151 |
| Ile151 | Amine (N) | 2.85 Å* | Carbonyl (O) | Gly114 |

* Symmetrical hydrogen bond distances are averaged.
