# Supplementary material for: Molecular recognition and maturation of SOD1 by its evolutionarily destabilised cognate chaperone hCCS
Source: PLoS Biol. 2019 Feb 8;17(2):e3000141. doi: 10.1371/journal.pbio.3000141 (PMC6383938; doi:10.1371/journal.pbio.3000141)
Supplement: S3 Table — hCCS, human copper chaperone for SOD1 (DOCX) [file pbio.3000141.s010.docx]

**S3 Table. hCCS domain II interface non-covalent bonding interactions.**

| **Amino Acid** | **Group** | **Distance** | **Group** | **Amino Acid** |
| --- | --- | --- | --- | --- |
|  | | | | |
| *Conserved from SOD1* | | | | |
| Gly135 | Amine (N) | 3.08 Å* | Carbonyl (O) | Arg232 |
| Arg232 | Amine (N) | 2.83 Å* | Carbonyl (O) | Gly195 |
|  | | | | |
| *Specific to hCCS* | | | | |
| Arg104 | Guanidinium (NH2) | 3.55 Å | Carboxylate (OD2) | Asp136 |
| Arg232 | Side chain amine (NE) | 3.04 Å* | Carbonyl (O) | Ile194 |
| Asp136 | Carboxylate (OD2) | 2.78 Å^#^ | Side chain OH (OG) | Ser233 |

* Symmetrical hydrogen bond distances are averaged over 1DO5 and the domain II structure presented here except those involving Arg104.

^#^ One Asp136 has different rotamer and does not interact.
