# Supplementary material for: Molecular recognition and maturation of SOD1 by its evolutionarily destabilised cognate chaperone hCCS
Source: PLoS Biol. 2019 Feb 8;17(2):e3000141. doi: 10.1371/journal.pbio.3000141 (PMC6383938; doi:10.1371/journal.pbio.3000141)
Supplement: S4 Table — hCCS, human copper chaperone for SOD1; SOD1, superoxide dismutase-1. (DOCX) [file pbio.3000141.s011.docx]

**S4 Table. hCCS-SOD1 complex interface non-covalent bonding interactions.**

| **Amino Acid** | **Group (atom)** | **Distance** | **Group (atom)** | **Amino Acid** |
| --- | --- | --- | --- | --- |
|  | | | | |
| **hCCS-SOD1 heterodimer interface (domain II-SOD1 only)** | | | | |
| *Conserved from SOD1* | | | | |
| SOD1 Gly51 | Amine (N) | 3.03 Å^&^ | Carbonyl (O) | hCCS Arg232 |
| SOD1 Ile151 | Amine (N) | 2.90 Å | Carbonyl (O) | hCCS Gly195 |
| SOD1 Ile151 | Carbonyl (O) | 2.72 Å | Amine (N) | hCCS Gly135 |
| SOD1 Gly114 | Carbonyl (O) | 2.83 Å | Amine (N) | hCCS Arg232 |
|  | | | | |
| *Specific to hCCS* | | | | |
| SOD1 Asp52 | Amine (N) | 3.25 Å | Carbonyl (O) | hCCS Arg232 |
| SOD1 Asp52 | Carboxylate (OD2) | 3.57 Å* | Guanidinium (NH1) | hCCS Arg104 |
| SOD1 Thr54 | Side-chain OH (OG1) | 3.61 Å | Guanidinium (NH2) | hCCS Arg104 |
| SOD1 Ile113 | Carbonyl (O) | 2.44 Å | Guanidinium (NE) | hCCS Arg232 |
|  | | | | |
| **Full-length hCCS-SOD1 heterodimer comprising compact and elongated structures** | | | | |
| *Conserved from SOD1* | | | | |
| SOD1 Gly51 | Amine (N) | 2.76 Å^&^ | Carbonyl (O) | hCCS Arg232 |
| SOD1 Ile151 | Amine (N) | 2.79 Å | Carbonyl (O) | hCCS Gly195 |
| SOD1 Ile151 | Carbonyl (O) | 2.82 Å | Amine (N) | hCCS Gly135 |
| SOD1 Gly114 | Carbonyl (O) | 2.89 Å | Amine (N) | hCCS Arg232 |
|  | | | | |
| *Specific to hCCS* | | | | |
| SOD1 Asp52 | Amine (N) | 3.20 Å | Carbonyl (O) | hCCS Arg232 |
| SOD1 Asp52 | Carboxylate (OD2) | 3.33 Å^#^ | Guanidinium (NH1) | hCCS Arg104 |
| SOD1 Thr54 | Side-chain OH (OG1) | 3.33 Å^+^ | Guanidinium (NH2) | hCCS Arg104 |
| SOD1 Ile113 | Carbonyl (O) | 3.01 Å | Guanidinium (NE) | hCCS Arg232 |

^&^ This discrepancy is due to hCCS Arg232 being the last residue in the domain II construct.

* 2 of 4 heterodimers in this P4_3_2_1_2 structure have Arg104-Asp52 distances of 3.35 Å. The remaining 2 heterodimers have a slight change in the Arg104 side chain which evens the distance between guanidinium N1 and N2 atoms and Asp52 side-chain carboxylate to 4.0 Å. This is likely due to the presence of symmetry related molecules over the salt-bridge.

^#^ Figure quoted is averaged over 12 and 2 full-length hCCS molecules in compact and elongated crystal forms respectively. Independent compact conformer average is 3.26 Å, independent elongated conformer average is 3.75 Å.

^+^ Figure quoted is averaged over 12 and 2 full-length hCCS molecules in compact and elongated crystal forms respectively. Independent compact conformer average is 3.27 Å, independent elongated conformer average is 3.70 Å.
