# Supplementary material for: Molecular recognition and maturation of SOD1 by its evolutionarily destabilised cognate chaperone hCCS
Source: PLoS Biol. 2019 Feb 8;17(2):e3000141. doi: 10.1371/journal.pbio.3000141 (PMC6383938; doi:10.1371/journal.pbio.3000141)
Supplement: S5 Table — hCCS, human copper chaperone for SOD1; SOD1, superoxide dismutase-1. (DOCX) [file pbio.3000141.s012.docx]

**S5 Table. Internal SOD1 disulphide sub-loop hydrogen bonding in the disulphide intact homodimeric state in comparison with the conformation present in heterodimeric complex with hCCS.**

| **Amino Acid** | **Group (atom)** | **Distance** | | **Group (atom)** | **Amino Acid** |
| --- | --- | --- | --- | --- | --- |
|  |  | SOD1 homodimer | SOD1  complex |  |  |
| **Conserved** |  |  |  |  |  |
| His48 | Imidazole (ND1) | 2.82 Å | 2.88 Å | Carbonyl (O) | Gly61 |
| Glu49 | Amine (N) | 2.90 Å | 2.85 Å | Carbonyl (O) | Pro62 |
| Phe50 | Amine (N) | 3.02 Å | 3.03 Å | Carbonyl (O) | Ala60 |
| Thr54 | Amine (N) | 2.89 Å | 3.34 Å | Carboxylate (OD1) | Asp52 |
| Thr54 | Side-chain OH (OG1) | 2.66 Å | 2.60 Å | Carboxylate (OD1) | Asp52 |
| Ala55 | Amine (N) | 2.81 Å | 2.60 Å | Side-chain OH (OG) | Ser59 |
| Ser59 | Side-chain OH (OG) | 3.42 Å | 2.95 Å | Carbonyl (O) | Gly56 |
|  |  |  |  |  |  |
| **Rearranged** |  |  |  |  |  |
| Ala60 | Amine (N) | 3.16 Å | N/A | Carbonyl (O) | Cys57 |
| Ala60 | Amine (N) | N/A | 3.24 Å | Carbonyl (O) | Gly56 |
|  |  |  |  |  |  |
| Gly61 | Amine (N) | 3.23 Å | N/A | Carbonyl (O) | Th58 |
| Gly61 | Amine (N) | N/A | 3.06 Å | Carbonyl (O) | Ala57* |
|  |  |  |  |  |  |
| **Lost** |  |  |  |  |  |
| Gly56 | Amine (N) | 3.03 Å | N/A | Carbonyl (O) | Asn53 |
| Ser59 | Amine (N) | 3.14 Å | N/A | Carbonyl (O) | Gly56 |
|  |  |  |  |  |  |
| **Gained** |  |  |  |  |  |
| Thr58 | Amine (N) | N/A | 3.30 Å | Carbonyl (O) | Ala55 |
| Ser59 | Side-chain OH (OG) | N/A | 3.05 Å | Carbonyl (O) | Asp52 |

*Ala57 substituted for Cys57.
