# Supplementary material for: Molecular recognition and maturation of SOD1 by its evolutionarily destabilised cognate chaperone hCCS
Source: PLoS Biol. 2019 Feb 8;17(2):e3000141. doi: 10.1371/journal.pbio.3000141 (PMC6383938; doi:10.1371/journal.pbio.3000141)
Supplement: S6 Table — hCCS, human copper chaperone for SOD1; SOD1, superoxide dismutase-1. (DOCX) [file pbio.3000141.s013.docx]

**S6 Table. The effect of SOD1 disulphide bond formation on hCCS-SOD1 inter-subunit non-covalent bonding interactions.**

| **Amino Acid** | **Group (atom)** | **Distance** | **Group (atom)** | **Amino Acid** |
| --- | --- | --- | --- | --- |
|  | | | | |
| *hCCS-SOD1 heterodimer interface (domain II-SOD1 only)* | | | | |
| SOD1 Gly51 | Amine (N) | 2.92 Å | Carbonyl (O) | hCCS Arg232 |
| SOD1 Ile151 | Amine (N) | 3.08 Å | Carbonyl (O) | hCCS Gly195 |
| SOD1 Ile151 | Carbonyl (O) | 2.97 Å | Amine (N) | hCCS Gly135 |
| SOD1 Gly114 | Carbonyl (O) | 2.65 Å | Amine (N) | hCCS Arg232 |
|  | | | | |
| SOD1 Asp52 | Amine (N) | 4.23 Å | Carbonyl (O) | hCCS Arg232 |
| SOD1 Asp52 | Carboxylate (OD2) | 4.45 Å | Guanidinium (NH1) | hCCS Arg104 |
| SOD1 Ile113 | Carbonyl (O) | 3.28 Å | Guanidinium (NE) | hCCS Arg232 |
|  |  |  |  |  |
| *Full-length hCCS-SOD1 heterodimer comprising compact and elongated structures.* | | | | |
| SOD1 Gly51 | Amine (N) | 2.80 Å | Carbonyl (O) | hCCS Arg232 |
| SOD1 Ile151 | Amine (N) | 2.89 Å | Carbonyl (O) | hCCS Gly195 |
| SOD1 Ile151 | Carbonyl (O) | 2.97 Å | Amine (N) | hCCS Gly135 |
| SOD1 Gly114 | Carbonyl (O) | 2.97 Å | Amine (N) | hCCS Arg232 |
|  | | | | |
| SOD1 Asp52 | Amine (N) | 3.73 Å | Carbonyl (O) | hCCS Arg232 |
| SOD1 Asp52 | Carboxylate (OD2) | 4.30 Å | Guanidinium (NH1) | hCCS Arg104 |
| SOD1 Thr54 | Side-chain OH (OG) | 4.46 Å | Guanidinium (NH2) | hCCS Arg104 |
| SOD1 Ile113 | Carbonyl (O) | 2.93 Å | Guanidinium (NE) | hCCS Arg232 |
